# Stimuli-Responsive DNA-Based Hydrogels on Surfaces for Switchable Bioelectrocatalysis and Controlled Release of Loads

**DOI:** 10.1021/acsami.3c06230

**Published:** 2023-07-21

**Authors:** Michael Fadeev, Gilad Davidson-Rozenfeld, Zhenzhen Li, Itamar Willner

**Affiliations:** The Institute of Chemistry, The Center for Nanoscience and Nanotechnology, The Hebrew University of Jerusalem, Jerusalem 91904, Israel

**Keywords:** pH, G-quadruplex, i-motif, stiffness, insulin, drug release, artificial pancreas

## Abstract

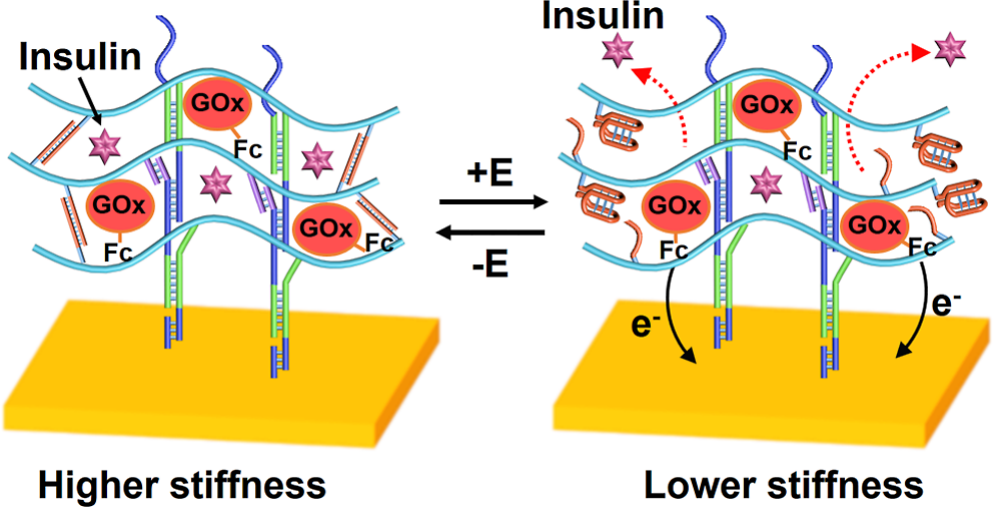

The assembly of enzyme [glucose oxidase (GOx)]-loaded stimuli-responsive DNA-based hydrogels on electrode surfaces, and the triggered control over the stiffness of the hydrogels, provides a means to switch the bioelectrocatalytic functions of the hydrogels. One system includes the assembly of GOx-loaded, pH-responsive, hydrogel matrices cross-linked by two cooperative nucleic acid motives comprising permanent duplex nucleic acids and “caged” i-motif pH-responsive duplexes. Bioelectrocatalyzed oxidation of glucose leads to the formation of gluconic acid that acidifies the hydrogel resulting in the separation of the i-motif constituents and lowering the hydrogel stiffness. Loading of the hydrogel matrices with insulin results in the potential-triggered, glucose concentration-controlled, switchable release of insulin from the hydrogel-modified electrodes. The switchable bioelectrocatalyzed release of insulin is demonstrated in the presence of ferrocenemethanol as a diffusional electron mediator or by applying an electrically wired integrated matrix that includes ferrocenyl-modified GOx embedded in the hydrogel. The second GOx-loaded, stimuli-responsive, DNA-based hydrogel matrix associated with the electrode includes a polyacrylamide hydrogel cooperatively cross-linked by duplex nucleic acids and “caged” G-quadruplex-responsive duplexes. The hydrogel matrix undergoes K^+^-ions/crown ether-triggered stiffness changes by the cyclic K^+^-ion-stimulated formation of G-quadruplexes (lower stiffness) and the crown ether-induced separation of the G-quadruplexes (higher stiffness). The hydrogel matrices demonstrate switchable bioelectrocatalytic functions guided by the stiffness properties of the hydrogels.

## Introduction

Stimuli-responsive hydrogel materials^1−4^ have attracted growing interest as “smart” materials for diverse applications, such as sensing,^5−9^ controlled drug release,^10−17^ tissue engineering,^18−22^ self-healing,^23−28^ shape-memory,^29−36^ robotics, and actuation.^37−44^ Different physical and chemical stimuli to trigger hydrogel materials and control their stiffness properties were introduced, including light,^45−50^ electrical fields,^51−54^ temperatures,^37,55−59^ magnetic fields,^12,60−64^ ultrasound irradiation,^65,66^ pH,^67−73^ and chemical agents.^74−79^ An important subclass of stimuli-responsive hydrogels includes biomaterial-based hydrogels^80−82^ and particularly, nucleic acid-based hydrogel matrices.^80−84^ The information encoded in the base sequence of nucleic acids provides a versatile means to cross-link the hydrogel matrices and to trigger the reversible reconfiguration of nucleic acid cross-linking bridging units, thereby controlling the stiffness of the hydrogel matrices. Indeed, within the family of nucleic acid-based stimuli-responsive hydrogels, all-DNA hydrogels^85,86^ or hybrid nucleic acid-functionalized polymer hydrogels^72,87^ can be identified. While all-DNA hydrogels consist of supramolecular oligonucleotide-entangled biopolymers^88,89^ or multi-dentate duplex-bridged Y-shaped or holliday-junction-bridged DNA structures,^72,85−87^ a hybrid polymer-DNA hydrogel can be cooperatively cross-linked by covalent cross-linking units or supramolecular bridging constituents and stimuli-responsive nucleic acid bridges^90^ or, alternatively, cooperatively cross-linked by permanent duplex or triplex cross-linking units and stimuli-reconfigurable nucleic acid bridges.^91^ Different chemical or physical triggers were applied to reconfigure nucleic acid-bridged hydrogels and reversibly control their stiffness. These included metal-ion co-stabilized duplex nucleic acid bridges, e.g., T-Hg^2+^-T or C-Ag^+^-C, and their separation by appropriate ligands, such as thiols,^92^ the reversible pH-stimulated formation and separation of i-motif structures^92^ or triplex bridges,^93^ the formation of G-quadruplexes and their separation in the presence of crown ether,^94^ the use of physical triggers, such as light, and the light-stimulated stabilization of duplex nucleic acids by *trans*-azobenzene intercalator units and the separation of the duplex bridges upon photoisomerization of the intercalators into the *cis*-azobenzene state^95^ or the thermoplasmonic separation of duplex nucleic acids by Au nanoparticles or Au nanorods.^96^ Diverse applications of stimuli-responsive DNA-based hydrogels were demonstrated, including controlled and switchable drug release,^95,97^ the use of the hydrogels as shape-memory,^29−36^ self-healing,^23−28^ and mechanically triggered matrices.^93,94^ Also, the stimuli-responsive hydrogels were employed as functional materials to construct gated microcarriers for the triggered release of drugs, such as DNA-based hydrogel microcapsules^98−100^ or hydrogel-coated metal–organic framework nanoparticles.^101^

The assembly of stimuli-responsive DNA-based hydrogels on surfaces is particularly challenging. Two general strategies to assemble stimuli-responsive hydrogel films on surfaces were introduced. One method involved the assembly of all-DNA hydrogels on surfaces using clamped hairpin DNA structures and the hybridization chain reaction.^102^ The second approach included the use of promoter nucleic acid-functionalized surfaces and the promoter-triggered assembly of nucleic acid-bridged hydrogel films by the cross-linking of two DNA hairpin-functionalized polymer chains, using the hybridization chain reaction.^103^ These methods were applied to pattern surfaces with DNA hybrids^102,104^ and to assemble hydrogels, exhibiting switchable stiffness properties on electrode supports, demonstrating switchable electrocatalysis.^103^

Here, we wish to report on the assembly of two different stimuli-responsive glucose oxidase (GOx)-loaded hydrogels on electrode surfaces. One system involves the assembly of pH-responsive DNA-based polyacrylamide hydrogel matrices. The GOx-catalyzed aerobic oxidation of glucose leads to acidification of the hydrogel matrix and accompanying changes in the hydrogel stiffness. By loading the hydrogel with tetramethylrhodamine-dextran (TMR-D) or insulin, the switchable biocatalyzed-stimulated pH changes, and accompanying stiffness changes of the hydrogels, are used for pH-triggered release of the loads. In addition, under anaerobic conditions, and by applying electron mediators to electrically wire GOx with the electrode surface, the bioelectrocatalyzed oxidation of glucose results in the acidification of the hydrogels and the potential-induced switchable stiffness changes of the hydrogel matrices, leading to the triggered release of the TMR-D or insulin loads. Beyond the significance of the systems demonstrating the biocatalyzed and bioelectrocatalyzed control over the stiffness of hydrogels by a metabolite (glucose), the systems could have significant applicability as autonomous electrical devices for controlled, glucose-guided, release of insulin for diabetic management. The second system involves the assembly of a GOx-loaded K^+^-ion/crown ether G-quadruplex-responsive DNA-polyacrylamide hydrogel on an electrode surface. By the reversible K^+^-ion and crown ether treatment of the hydrogel, it is switched between lower and higher stiffness states. This allows the control over the electrical wiring efficiency between GOx and the electrode support.

## Results and Discussion

**Figure 1:**
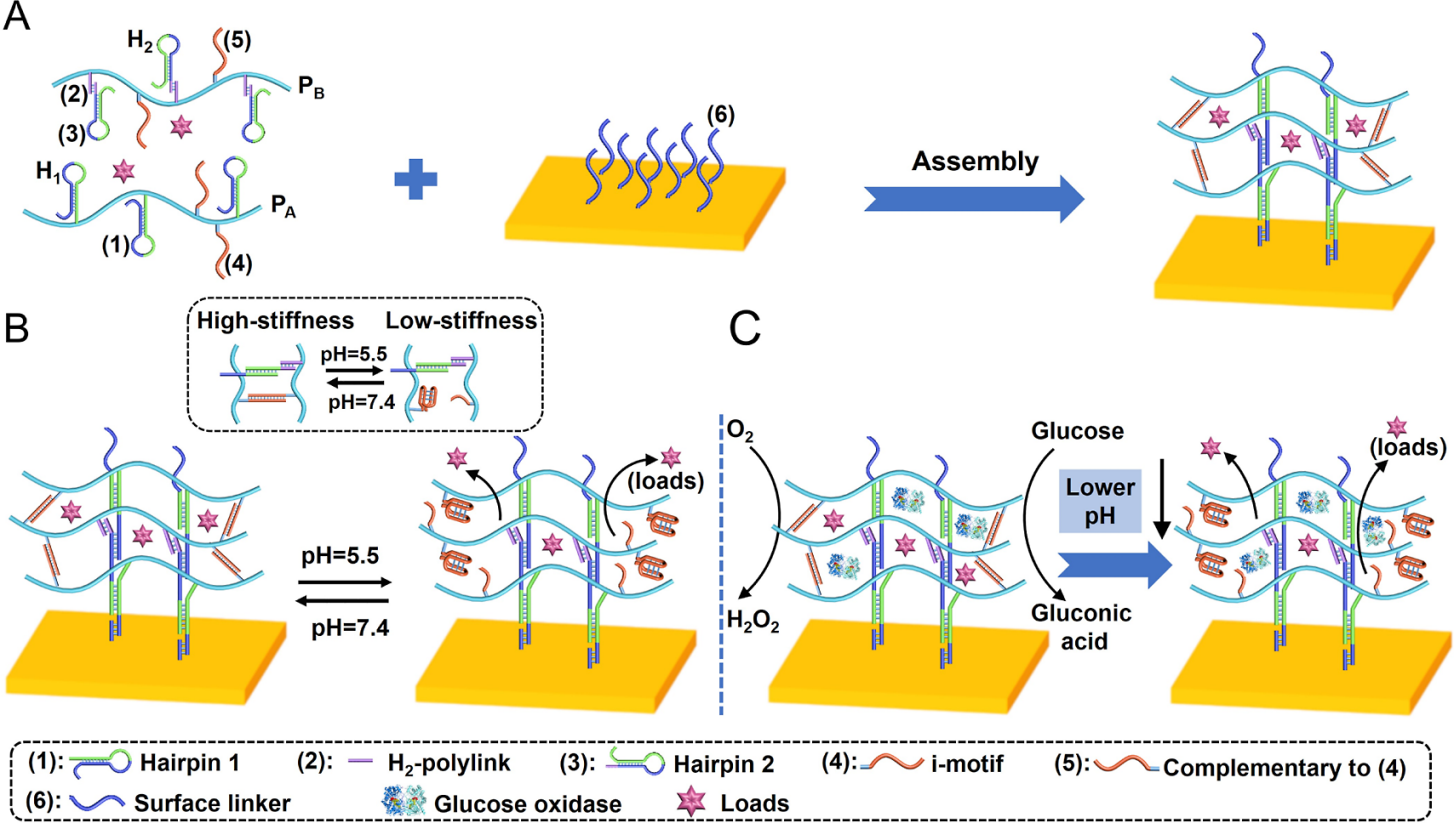
(A) Schematic assembly of loaded pH-responsive DNA hydrogels on Au-coated surfaces (loads: TMR-D or coumarin-labeled insulin). (B) Switchable pH-induced release of loads by auxiliary pH triggers. (C) Hydrogel loaded with glucose oxidase (GOx) undergoing pH-induced release of the loads through the aerobic GOx-biocatalyzed oxidation of glucose. The release of the loads is stimulated by the pH-triggered control over the stiffness of the hydrogel through the separation of the (**4**)/(**5**) duplexes into an i-motif configuration.

Figure 1A depicts the method to assemble the pH-responsive glucose oxidase (GOx)-loaded hydrogel on the Au surface. Two nucleic acid-functionalized polyacrylamide chains, P_A_ and P_B_, were prepared. Polymer chain P_A_ is functionalized with hairpin H_1_, (**1**), and the single strand (**4**). Polymer P_B_ consists of a polyacrylamide chain functionalized with hairpin H_2_, (**3**), hybridized with the tether nucleic acid (**2**), directly conjugated to the polymer, and the single nucleic acid (**5**) is tethered to the polymer chain. The tethers (**4**) and (**5**) exhibit complementarity, and the sequence comprising the tether (**4**) is cytosine rich and reconfigures under acidic conditions, pH ≤ 6, into the i-motif structure. The hairpins H_1_ and H_2_ reveal cross-opening features and the opening of hairpin H_1_ allows the cross-opening of H_2_ and the open hairpin H_2_ allows the counter opening of H_1_, and vice versa. The loading of hairpin H_1_ (**1**) and the tether (**4**) on polymer P_A_ corresponded to 1:150 and 1:50 acrylamide monomer units. The loading of the polymer chain P_B_ with H_2_ and (**2**)/(**3**) complex and the tether (**5**) corresponded to 1:147 and 1:49 acrylamide units, respectively. (For the synthesis of polymer chains P_A_ and P_B_, their spectroscopic characterization, and the evaluation of the loading degrees of the respective nucleic acid units, see Supporting Information, PS4–PS7). To assemble the hydrogel on the Au surface, the Au surface was functionalized with the nucleic acid strand (**6**), with a surface coverage of ca. 25 pmol·cm^–2^, acting as a promoter strand to activate the cross-opening of the hairpins H_1_ and H_2_ associated with the polymer chains. Aqueous buffer droplet, 15 μL, that included each of the polymer chains P_A_ and P_B_, 7.5 μM, was deposited on the Au surface. Whenever the integration of GOx in the hydrogel was needed, GOx, 27 units, was included in the polymer mixture. Also, whenever the loading of the hydrogels was needed, the dye load (3 μL TMR-dextran, 25 mg/mL) or the insulin load (3 μL coumarin-functionalized insulin, 16 mM) were included in the polymer chain solution mixture. The promoter units, associated with the surface, activated the hybridization chain reaction (HCR), where the promoter strands opened hairpin H_1_ associated with P_A_, the opened H_1_ opened hairpin H_2_ associated with P_B_, and the open H_2_ units opened the H_1_ hairpin units associated with P_A_, and vice versa. This process resulted in the formation of a stiff hydrogel matrix (1000 μm thick), firmly fixed on the surface, of entangled P_A_ and P_B_ polymer chains, cooperatively cross-linked by duplexes (**1**)/(**2**) + (**3**), originating from the cross-opening of H_1_/H_2_, and the stimuli-responsive duplexes of tethers (**4**)/(**5**). Whenever GOx or the respective loads are included in the polymer mixture, the proteins/loads are physically immobilized in the hydrogel matrix. The mechanism of the pH-controlled transitions of the hydrogel between stiff and lower stiffness states by auxiliary pH changes or through the biocatalyzed oxidation of glucose, and the pH-stimulated release of the respective integrated loads, is schematically presented in Figure 1B,C. Subjecting the hydrogel matrix to pH = 5.5 leads to the reconfiguration of the strands (**4**) into the i-motif structures and to the separation of the duplex nucleic acid bridges (**4**)/(**5**). This results in the removal of one of the cross-linking motives and to the formations of a lower stiffness hydrogel that allows the release of the load. The neutralization of the hydrogel separates the i-motif structures and regenerates the duplex bridges (**4**)/(**5**), the formation of the stiff hydrogel and the blockage (switch-off state) of the release of the loads. In the presence of glucose and GOx, and in the absence of auxiliary acidification of the hydrogel matrix, the aerobic GOx-catalyzed oxidation of glucose by oxygen in the hydrogel matrix yields gluconic acid, as shown in eq 1. The local acidification of the hydrogel matrix leads to the separation of the duplex bridging units (**4**)/(**5**) through the formation of the i-motif structures and to the formation of the lower stiffness hydrogel. This allows the release of the load (e.g., insulin). As the pH changes in the hydrogel matrix are controlled by concentrations of glucose, the efficacy of the release of the load (e.g., insulin) is guided by the concentrations of glucose. The loading of GOx in the GOx-containing hydrogel was evaluated by assaying the activity of the immobilized enzyme, and it corresponds to 3.22 units/mm^3^ (for details see Supporting Information, PS8).
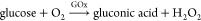
1

**Figure 2:**
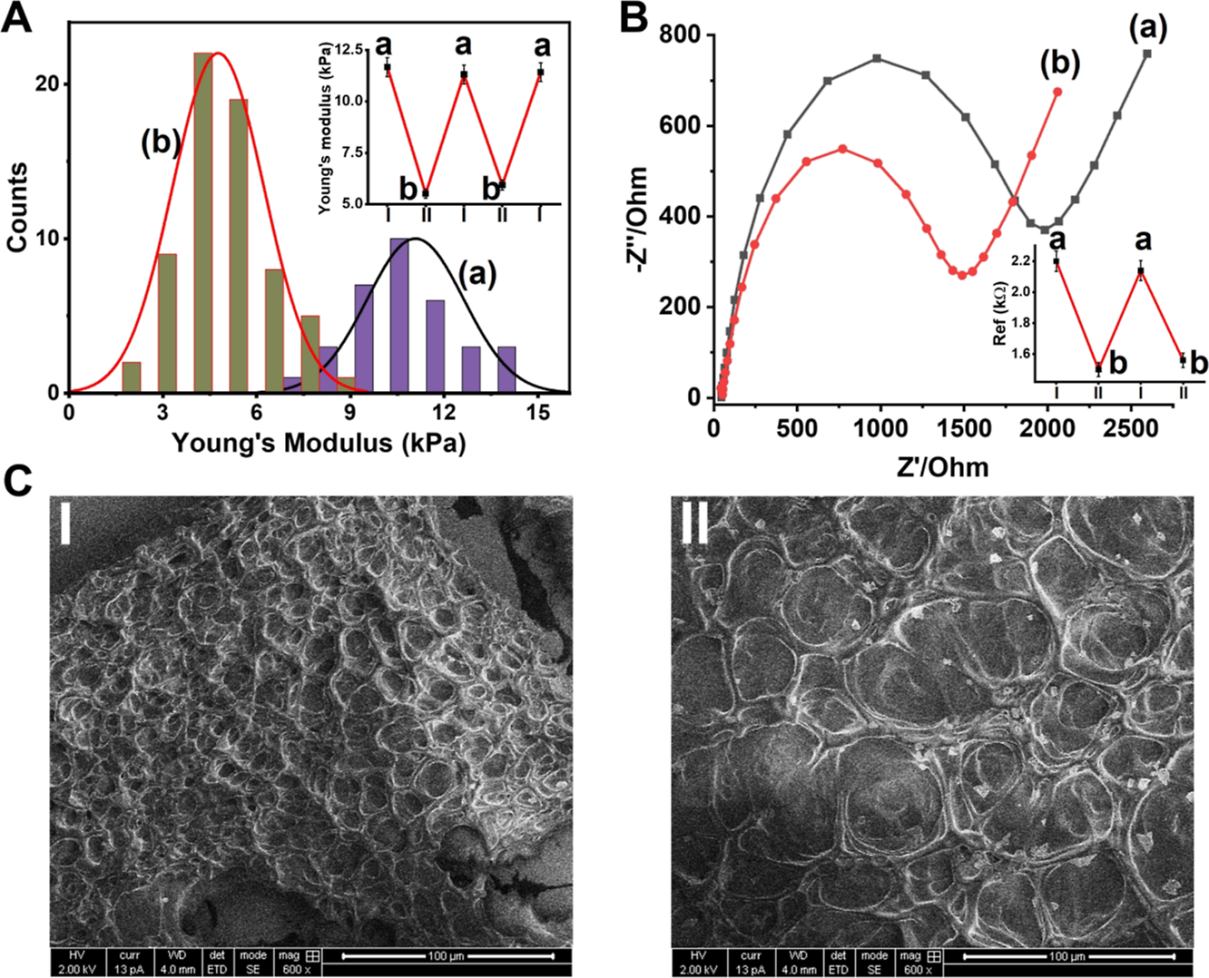
(A) Young’s modulus histograms corresponding to (a) higher stiffness hydrogel, at pH = 7.4, cross-linked by (**1**)/(**2**) + (**3**) and the duplexes (**4**)/(**5**). (b) Lower stiffness hydrogel at pH = 5.5 cross-linked by only (**1**)/(**2**) + (**3**). Inset: switchable Young’s moduli of the pH-responsive hydrogel by subjecting the hydrogel to pH = 7.4 (a) and pH = 5.5 (b). (B) Faradaic impedance spectra corresponding to: (a) higher stiffness hydrogel cross-linked by (**1**)/(**2**) + (**3**) and (**4**)/(**5**) at pH = 7.4. (b) Lower stiffness hydrogel cross-linked by (**1**)/(**2**) + (**3**) at pH = 5.5. Inset: Switchable interfacial electron transfer resistances of (a) higher stiffness hydrogel at pH = 7.4. (b) Lower stiffness hydrogel at pH = 5.5. (C) Scanning electron microscopy images of: Panel I—the higher stiffness hydrogel associated with the surface, pH = 7.4, and Panel II—the lower stiffness hydrogel, pH = 5.5, associated with the surface. Error bars evaluated from *N* = 3 experiments.

The stiffness properties and porosity of the pH-responsive hydrogel were examined in the absence of GOx or in the presence of GOx/glucose-loaded hydrogel. Figure 2A shows the histograms evaluating the Young’s moduli of the pH-responsive hydrogel (lacking immobilized GOx) upon subjecting the matrix to auxiliary pH changes: pH = 7.4 or pH = 5.5, using a microindentation technique. The Young’s modulus of the hydrogel matrix at pH = 7.4 corresponds to 11 kPa, whereas the Young’s modulus of the hydrogel at pH = 5.5 corresponds to ca. 5 kPa, implying that the hydrogel at pH = 7.4 reveals higher stiffness as compared to pH = 5.5. This result is consistent with the fact that at pH = 7.4 the hydrogel is cross-linked by two cooperative motives: the duplexes (**1**)/(**2**) + (**3**) and the pH-responsive duplexes (**4**)/(**5**), leading to the higher stiffness hydrogel. At pH = 5.5, the duplex units (**4**)/(**5**) are separated, through the reconfiguration of (**4**) into i-motif structures, leading to a lower stiffness hydrogel. The control over the stiffness is reversible and by switching the pH of the hydrogel environments between pH = 7.4 and pH = 5.5, the hydrogel is cycled between higher and lower stiffness values, as shown in Figure 2A, inset. Figure 2B shows the Faradaic impedance spectra of the hydrogel-functionalized Au electrode at pH = 7.4, curve (a), and at pH = 5.5, curve (b), using Fe(CN)_6_^3–/4–^ as redox probe. The interfacial electron-transfer resistance of the hydrogel at pH = 7.4 corresponds to *R*_et_ = 2.6 kΩ, whereas the electron-transfer resistance of the hydrogel at pH = 5.5 is lower, *R*_et_ = 1.7 kΩ. These results are consistent with the higher stiffness and lower permeability of the redox probe into the cooperatively cross-linked hydrogel at pH = 7.4, leading to higher interfacial electron-transfer resistance.^104^ For the application of time-dependent Faradaic impedance spectra to follow the dynamic transitions of the hydrogel between the high-stiffness hydrogel and the low-stiffness hydrogel, see Figure S7A,B. The switchable electron-transfer resistances of the electrode are reversible upon cycling the pH between the values pH = 7.4 and pH = 5.5, as shown in Figure 2B, inset. Scanning electron microscopy measurements further support the porosity and permeability properties of the pH-responsive hydrogel. Figure 2C shows characteristic SEM images of the hydrogel matrix at pH = 7.4, panel I, and pH = 5.5, panel II. At pH = 7.4, a dense array of small pores is observed, while at pH = 5.5 substantially larger pores are observed. These results are consistent with the higher degree of cross-linking and enhanced stiffness of the hydrogel at pH = 7.4.

**Figure 3:**
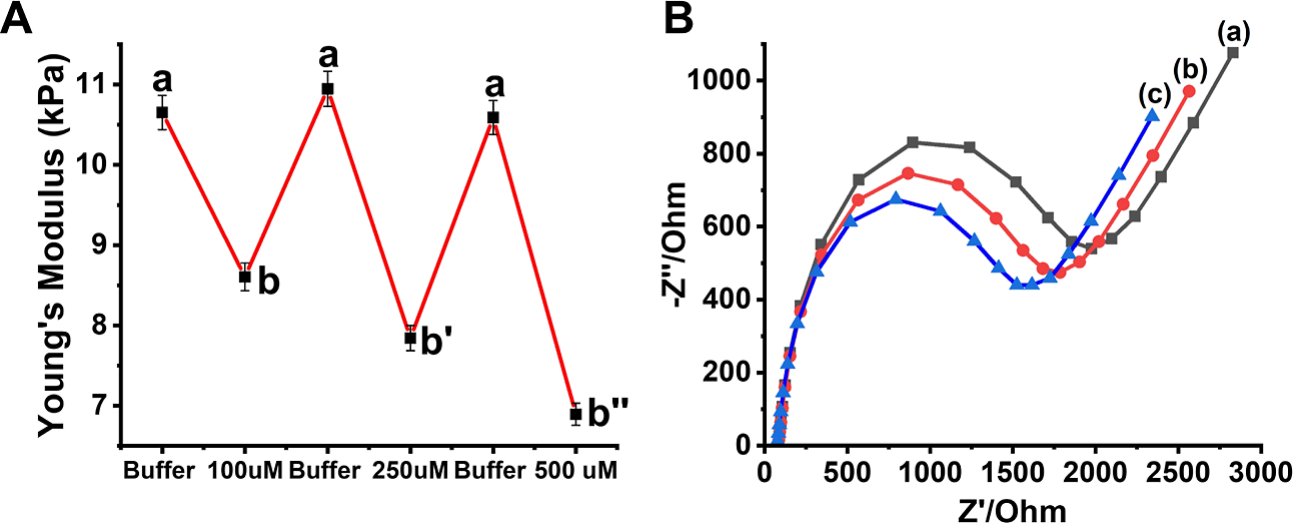
(A) Switchable Young’s moduli upon cyclic treatment of the GOx-loaded higher stiffness hydrogel-modified surface (a) with variable concentrations of glucose: (b) 100 μM, (b′) 250 μM, and (b″) 500 μM. (B) Faradaic impedance spectra corresponding to the GOx-loaded hydrogel: (a) in the absence of added glucose; (b) in the presence of glucose 50 μM; and (c) in the presence of glucose 250 μM. The GOx-biocatalyzed oxidation of glucose allowed to proceed for a time-interval corresponding to 15 min. The microindentations and the electrochemical experiments were performed in HEPES buffer (10 mM, pH = 7.4, NaCl 50 mM, and MgCl_2_ 5 mM). Error bars evaluated from *N* = 3 experiments.

The control over the stiffness properties of the GOx-loaded stimuli-responsive hydrogel through the GOx-biocatalyzed aerobic oxidation of glucose is presented in Figure 3. The Young’s moduli of the GOx-loaded pH-responsive hydrogel subjected to variable concentrations of glucose for a fixed time interval of 15 min are displayed in Figure 3A. Treatment of the GOx-functionalized pH-responsive hydrogel-modified electrode with 100 μM glucose resulted in the decrease of the Young’s moduli from 10.6 to 8.5 kPa, which is consistent with the biocatalyzed formation of gluconic acid, acidification of the hydrogel matrix, and the separation of the (**4**)/(**5**) stimuli-responsive duplex. Rinsing the glucose-treated hydrogel with a buffer solution, pH = 7.4, restores the higher stiffness hydrogel, Young’s modulus 10.6 kPa, which is consistent with the separation of the i-motif units and the regeneration of the hydrogel cross-linked by (**1**)/(**2**) + (**3**) and (**4**)/(**5**). Repeated treatment of the hydrogel with 250 μM glucose and 500 μM glucose for a time interval of 15 min resulted in the decrease of the Young’s moduli of the hydrogel to 7.6 and 6.8 kPa, respectively. As the concentration of glucose increases, the biocatalyzed generation of gluconic acid and the separation of the (**4**)/(**5**) are enhanced, resulting in lower stiffness values of the hydrogel. The GOx-guided biocatalyzed oxidation of glucose and the control over the stiffness/porosity of the hydrogel are further supported by Faradaic impedance measurements, as shown in Figure 3B. Upon increasing the concentration of glucose, the interfacial electron-transfer resistances of the hydrogel-modified surfaces decrease, which is consistent with the enhanced biocatalyzed acidification of the hydrogel and the glucose-controlled decrease of the stiffness and higher porosity of the hydrogel’s matrices. It should be noted that the Young’s modulus value could be switched upon being subjected to glucose and rinsing with the buffer solution for, at least, six cycles, as shown in Figure S8. Nevertheless, the mechanical degradation of the hydrogel framework upon the indentation process decreases the switching efficiency upon increasing the number of switching cycles.

### Biocatalyzed Release of Loads from the pH-Responsive Hydrogels

**Figure 4:**
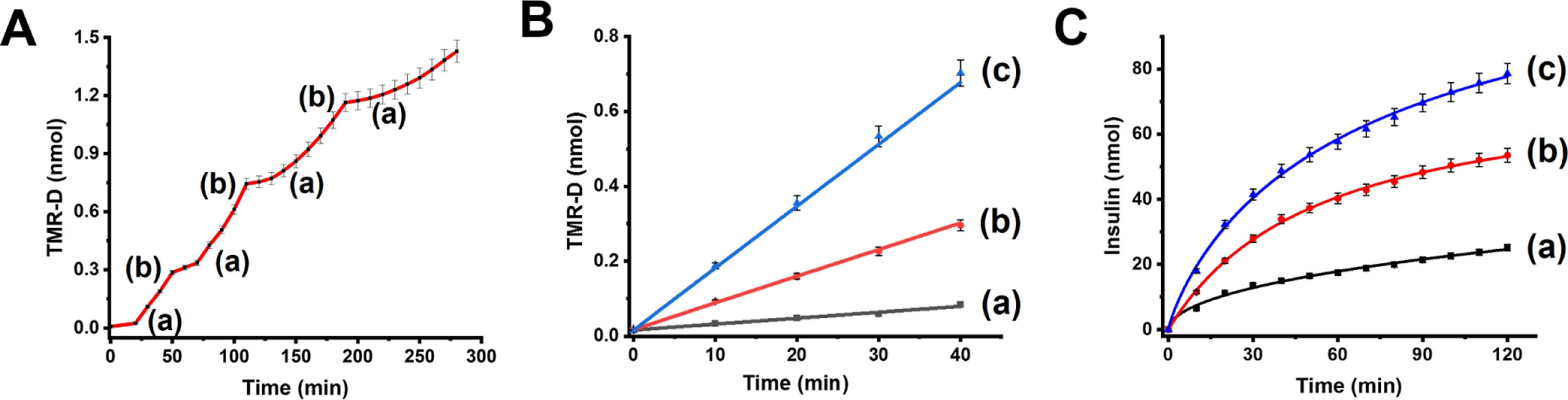
(A) Switchable, time-dependent, glucose-triggered release of TMR-D from the GOx-loaded pH-responsive hydrogel. At time-intervals marked with (a) hydrogel is subjected to glucose 250 μM, resulting in the triggered release of TMR-D. At time-intervals marked with (b), hydrogel is rinsed with a buffer solution, pH = 7.4, resulting in the blockage of TMR-D release. (B) Time-dependent release of TMR-D from the GOx-loaded pH-responsive hydrogel subjected to different concentrations of glucose: (a) 50; (b) 100; and (c) 250 μM. (C) Time-dependent release of coumarin-labeled insulin from the GOx/insulin-loaded pH-responsive hydrogel treated with different concentrations of glucose: (a) 50; (b) 100; and (c) 250 μM. Error bars evaluated from *N* = 4 experiments.

The control over the stiffness of the pH-responsive hydrogel by means of the GOx-catalyzed oxidation of glucose was, then, applied for the biocatalyzed release of loads from the hydrogel. As loads, we used the dye tetramethylrhodamine-dextran, TMR-D, or coumarin-labeled insulin. Figure 4A shows the stepwise GOx/glucose-stimulated release of TMR-D from the GOx-functionalized hydrogel loaded with TMR-D (GOx = 27 units; TMR-D loading = 1.9 nmol). At pH = 7.4, and in the absence of glucose, no TMR-D release from the hydrogel could be observed within a time interval of 12 h, indicating that the stiff hydrogel cooperatively stabilized by (**1**)/(**2**) + (**3**) and (**4**)/(**5**) duplexes does not allow the non-triggered leakage of TMR-D from the hydrogel. Treatment of the GOx biocatalyst/TMR-D-loaded matrix with glucose, 250 μM, as shown in Figure 4A, point (a), results in the time-dependent release of TMR-D. That is, the biocatalyzed oxidation of glucose acidifies the hydrogel, resulting in the separation of the (**4**)/(**5**) duplexes and the formation of the lower stiffness hydrogel that allows the release of TMR-D. After 30 min of releasing the load, the hydrogel was rinsed with a pure buffer solution, pH = 7.4, point (b), resulting in the blockage of the TMR-D release, where the recovery of the stiff hydrogel cooperatively stabilized by the duplexes (**1**)/(**2**) + (**3**) and the (**4**)/(**5**) prevents the release of the load. Re-addition of glucose, 250 μM, point (a), reactivates the release of TMR-D. By repeatedly rinsing off the glucose solution, the hydrogel matrix was re-blocked toward the release of TMR-D and by the re-addition of glucose, the release process was re-activated. The release rate of TMR-D was controlled by the concentration of glucose, as shown in Figure 4B. As the concentration of glucose was higher, the release rate was faster, which is consistent with the glucose-regulated control over the stiffness of the hydrogel. That is, as the concentration of glucose is higher, the pH changes stimulated by the aerobic GOx-catalyzed oxidation of glucose are intensified, resulting in higher degree of i-motif uncaging of the hydrogel, lowering the stiffness of hydrogel, accompanied by the enhanced release of the load. With the vision that biocatalyzed, glucose-controlled, release of loads from the pH-responsive hydrogel could provide a functional matrix for the controlled release of drugs, we attempted to apply the stimuli-responsive hydrogel as a functional matrix for the glucose-guided release of insulin. Accordingly, insulin was labeled with the coumarin fluorophore and loaded in the pH-responsive GOx-loaded matrix associated with the Au surface. Figure 4C depicts the time-dependent release of the coumarin-labeled insulin in the presence of different concentrations of glucose. As the concentration of glucose increases, the rates of release of the insulin are higher, which is consistent with the glucose-regulated pH changes and degree of i-motif uncaging of the cross-linked hydrogel matrix.

### Bioelectrocatalyzed Release of Loads from the pH-Responsive Hydrogel Matrix

The previous section demonstrated the pH-controlled release of loads from the hydrogel matrix driven by the aerobic GOx-catalyzed oxidation of glucose. A major effort in the area of bioelectrochemistry involves, however, the electrochemical activation of redox proteins, particularly, the activation of glucose oxidase, GOx, toward the oxidation of glucose, in the absence of oxygen, as shown in eq 2. While redox proteins, usually, lack direct electrochemical communication between their redox centers and electrode surfaces, the development of means to electrically communicate between the redox centers of proteins and the conductive support has attracted substantial research efforts.^105−109^ Diverse approaches to “electrically wire” redox proteins and electrodes were reported, including the application of diffusional electron mediators,^110,111^ the functionalization of the redox proteins with redox mediators,^112,113^ the wiring of redox proteins by means of electron transporting nanomaterials, such as Au-nanoparticles^114,115^ or carbon nanotubes,^116,117^ the reconstitution of apo-proteins with cofactor-relay conjugates,^118,119^ and the application of redox-modified soft polymer matrices loaded with the redox proteins.^120−125^ The methods to electrically wire redox protein with electrode supports were broadly employed for the development of electrochemical biosensors, especially glucose sensors.^124,126^ The electrical activation of glucose oxidase, as shown in eq 2, provides, however, a means to generate pH changes (acidification of the reaction medium) through the bioelectrocatalyzed oxidation of glucose. Accordingly, we argued that the bioelectrocatalyzed oxidation of glucose by the GOx-functionalized pH-responsive hydrogel could provide a means to control the stiffness of the hydrogel matrix and to stimulate the electrically-driven release of loads from the hydrogel. The development of the glucose-controlled electrochemically-driven release of loads, particularly of insulin, by an electrochemically active hydrogel-modified electrode may have important practical applications since such hydrogel-modified electrodes could, in principle, act as sense-and-treat devices for the glucose-controlled release of insulin.
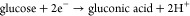
2

**Figure 5:**
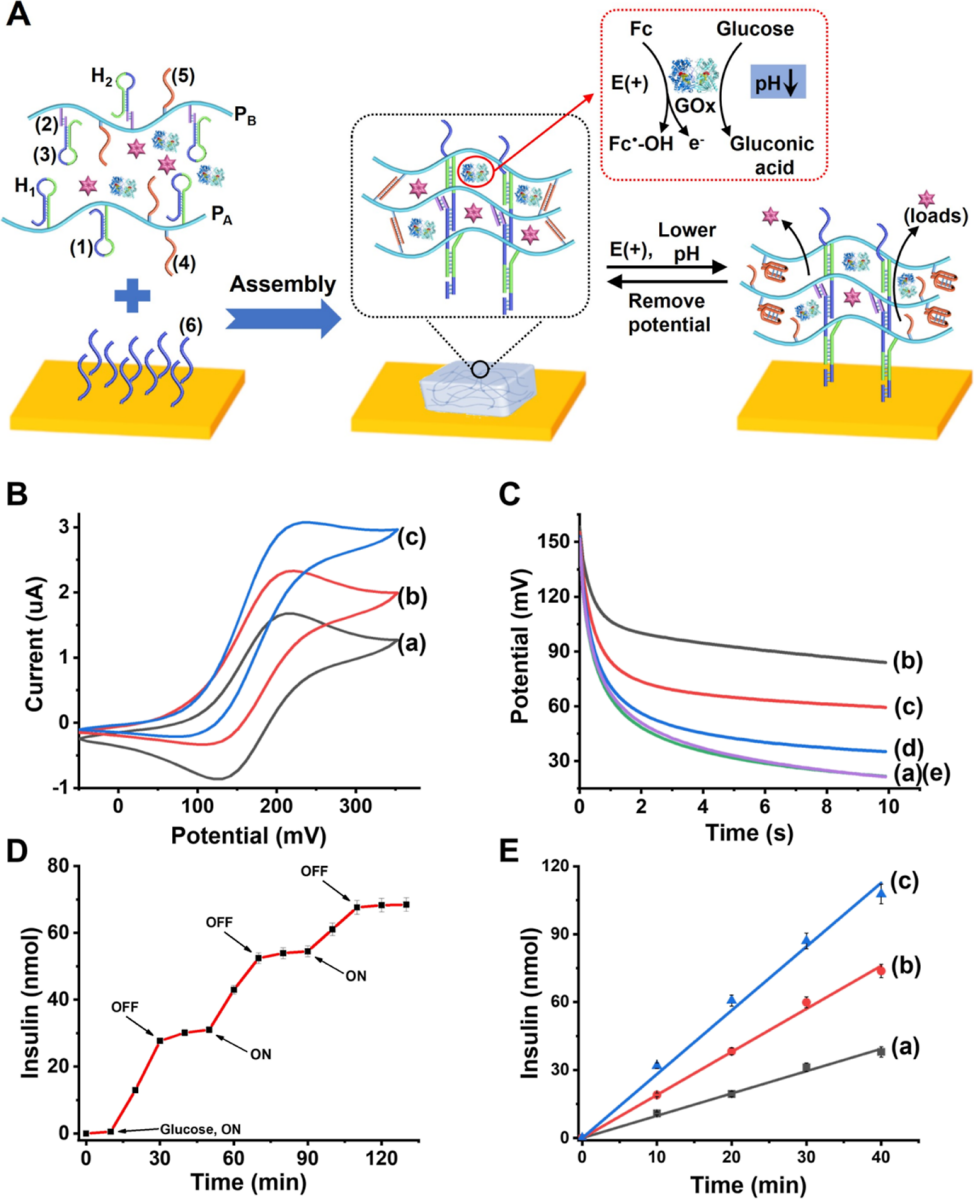
(A) Schematic assembly of the GOx-functionalized TMR-D or coumarin-labeled insulin-loaded in the pH-responsive hydrogel-modified Au-coated electrode, and schematic ferrocenemethanol-mediated bioelectrocatalyzed oxidation of glucose resulting in the acidification of the hydrogel, lowering the stiffness of the hydrogel, and the release of the loads. Upon switching off the potential on the electrode, the bioelectrocatalytic oxidation of glucose is switched-off, resulting in the pH-stimulated equilibration with the bulk electrolyte solution, pH = 7.4, leading to the recovery of the higher stiffness hydrogel where the release of the loads is switched-off. (B) Cyclic voltammograms generated by the GOx-loaded pH-responsive hydrogel-modified electrode, in the presence of ferrocenemethanol, in the absence of added glucose, curve (a), and in the presence of added glucose 100 μM, curve (b), and 250 μM, curve (c). (C) Chronopotentiometric transients corresponding to the GOx-loaded hydrogel matrix in the presence of ferrocenemethanol, 40 μM, glucose, 2 mM, under anaerobic conditions, constant current 10 μA. (a) Hydrogel matrix cross-linked by the (**1**)/(**2**) + (**3**) and (**4**)/(**5**) bridges prior to subjecting the electrode to the bioelectrocatalyzed oxidation of glucose. (b) After driving the bioelectrocatalyzed oxidation of glucose for 5 min (applied potential, 0.35 V vs SCE). (c) After allowing the electrode to rest for 20 s without any potential. Curves (d) and (e), allowing the electrode to rest for 50 and 90 s, respectively, without auxiliary potential. (D) Switchable, potential-induced, time-dependent release of the coumarin-labeled insulin load from the GOx-functionalized pH-responsive hydrogel in the presence of glucose 100 μM. Subjecting the potential, corresponding to 0.35 V vs SCE, on the electrode switches-on the bioelectrocatalyzed oxidation of glucose, acidification of the hydrogel, and the release of coumarin-labeled insulin. (E) Time-dependent release of coumarin-labeled insulin from the hydrogel carrier upon applying a fixed potential of 0.35 V vs SCE on the hydrogel-functionalized electrode, in the presence of variable glucose concentrations: (a) 50, (b) 100, and (c) 250 μM. Error bars evaluated from *N* = 4 experiments.

The GOx-loaded pH-responsive hydrogel-modified electrode, as shown in Figure 5A, cross-linked by the permanent duplexes (**1**)/(**2**) + (**3**) and the pH-responsive (**4**)/(**5**) duplexes was subjected under anaerobic conditions to the bioelectrocatalyzed oxidation of glucose in the presence of ferrocenemethanol, (**9**), as a diffusional electron mediator. Figure 5B shows the cyclic voltammograms of the GOx-loaded hydrogel in the presence of the ferrocenemethanol electron mediator (20 μM), in the absence of glucose, curve (a), and in the presence of added glucose, curves (b) and (c). While in the absence of glucose, the quasi-reversible redox wave of the electron mediator is observed, the addition of glucose results in electrocatalytic anodic currents that are intensified as the concentration of glucose increases. The progress of the GOx-electrocatalyzed oxidation of glucose suggests that the process leads to the acidification of the hydrogel matrix and to the electrochemically driven control of the stiffness of the hydrogel as a result of the bioelectrocatalyzed oxidation of glucose. Chronopotentiometric experiments,^127^ as shown in Figure 5C, support the pH-stimulated stiffness changes of the GOx-loaded (**1**)/(**2**) + (**3**) and (**4**)/(**5**) cross-linked hydrogel upon the ferrocenemethanol (**9**)-mediated bioelectrocatalyzed oxidation of glucose. Figure 5C, curve (a), depicts the chronopotentiometric curve of the GOx-loaded dual cross-linked hydrogel in the presence of glucose and the electron mediator prior to the triggered activation of the bioelectrocatalytic process. Realizing that the chronopotentiometric curve is recorded at a constant current of 10 μA, the interfacial electron-transfer resistance of the hydrogel matrix is estimated to be ca. *R*′ ≈ 12 kΩ. Subjecting the GOx-loaded hydrogel to the bioelectrocatalytic process for 5 min under constant potential (*E* = 0.35 V vs SCE) results in the chronopotentiometric curve shown in curve (b). The electron-transfer resistance of the electron is substantially lower as compared to the original value of the dual cross-linked hydrogel matrix (ca. *R*′ ≈ 6 kΩ). This result is consistent with the lowering of the pH of the hydrogel matrix, separation of the duplex bridges (**4**)/(**5**), and lowering of the stiffness of the hydrogel matrix. Subsequently, allowing the GOx-loaded hydrogel matrix that drives the bioelectrocatalyzed oxidation of glucose to rest, in the absence of any applied potential, while recording the temporal electron-transfer resistance of the hydrogel matrix by chronopotentiometry resulted in the chronopotentiometric responses depicted in curves (c), (d), and (e). Thus, within 90 s, the original electron-transfer resistance of the hydrogel matrix was recorded. That is, the local acidic pH within the hydrogel film was equilibrated by the neutral pH of the electrolyte solution that resulted in the recovery of the dual cross-linked high-stiffness hydrogel matrix. The control over the stiffness of the hydrogel by the GOx-bioelectrocatalyzed oxidation of glucose, and the accompanying acidification of the hydrogel was, then, used for the electrochemically triggered release of loads encapsulated in the hydrogel. The TMR-D load was integrated in the hydrogel matrix. Figure S12 in the Supporting Information depicts the stepwise potential-induced release of TMR-D from the hydrogel matrix. The application of a potential corresponding to 0.35 V vs SCE results in the oxidation of the electron mediator and the activation of the bioelectrocatalyzed oxidation of glucose, resulting in the acidification of the hydrogel matrix, the separation of the duplexes (**4**)/(**5**) by forming i-motif structures, and the transformation of the hydrogel into a matrix of lower stiffness that facilitates the release of TMR-D. Switching off the potential on the electrode blocked the bioelectrocatalytic process, and the localized acidic conditions associated with the hydrogel were depleted by proton diffusion into the bulk electrolyte. This resulted in the separation of the i-motif units and the reassembly of the higher stiffness (**4**)/(**5**) cross-linked hydrogel that blocked the release of TMR-D. By the ON/OFF-switched potential-induced activation/deactivation of the bioelectrocatalytic process, the release of the TMR-D load from the hydrogel was reversibly switched between “ON” and “OFF” states, as shown in Figure S12, Supporting Information. The method was, then, applied for the potential-stimulated ON/OFF release of coumarin-labeled insulin from the pH-responsive hydrogel, as shown in Figure 5D. The ferrocene-mediated bioelectrocatalyzed oxidation of glucose acidified the hydrogel and resulted in the formation of a lower stiffness hydrogel through the separation of the (**4**)/(**5**) cross-linking bridges that facilitated the release of coumarin-labeled insulin. Removal of the potential, oxidizing the electron mediator, from the electrode, blocked the bioelectrocatalyzed oxidation of glucose, leading to the neutralization of the pH and the recovery of the stiff (**4**)/(**5**)-duplex-bridged hydrogel that switched off the release of the insulin load. By the cyclic electrochemical activation and deactivation of the bioelectrocatalytic process, the release of insulin was switched between “ON” and “OFF” states. The rate of the release of the coumarin-labeled insulin from the hydrogel is controlled by the concentrations of glucose, as shown in Figure 5E. As the concentration of glucose increases, the rate of insulin release is enhanced. These results are consistent with the glucose-dictated pH-controlled stiffness of the hydrogel matrix. As the concentration of glucose increases, the bioelectrocatalyzed oxidation of glucose is enhanced, resulting in higher local pH changes (acidification) of the hydrogel matrix. That is, as the concentration of glucose increases, the degree of unlocking of the hydrogel matrix, through separation of the (**4**)/(**5**) bridges, is higher, resulting in lower stiffness of the hydrogel and enhanced rates of release of insulin.

**Figure 6:**
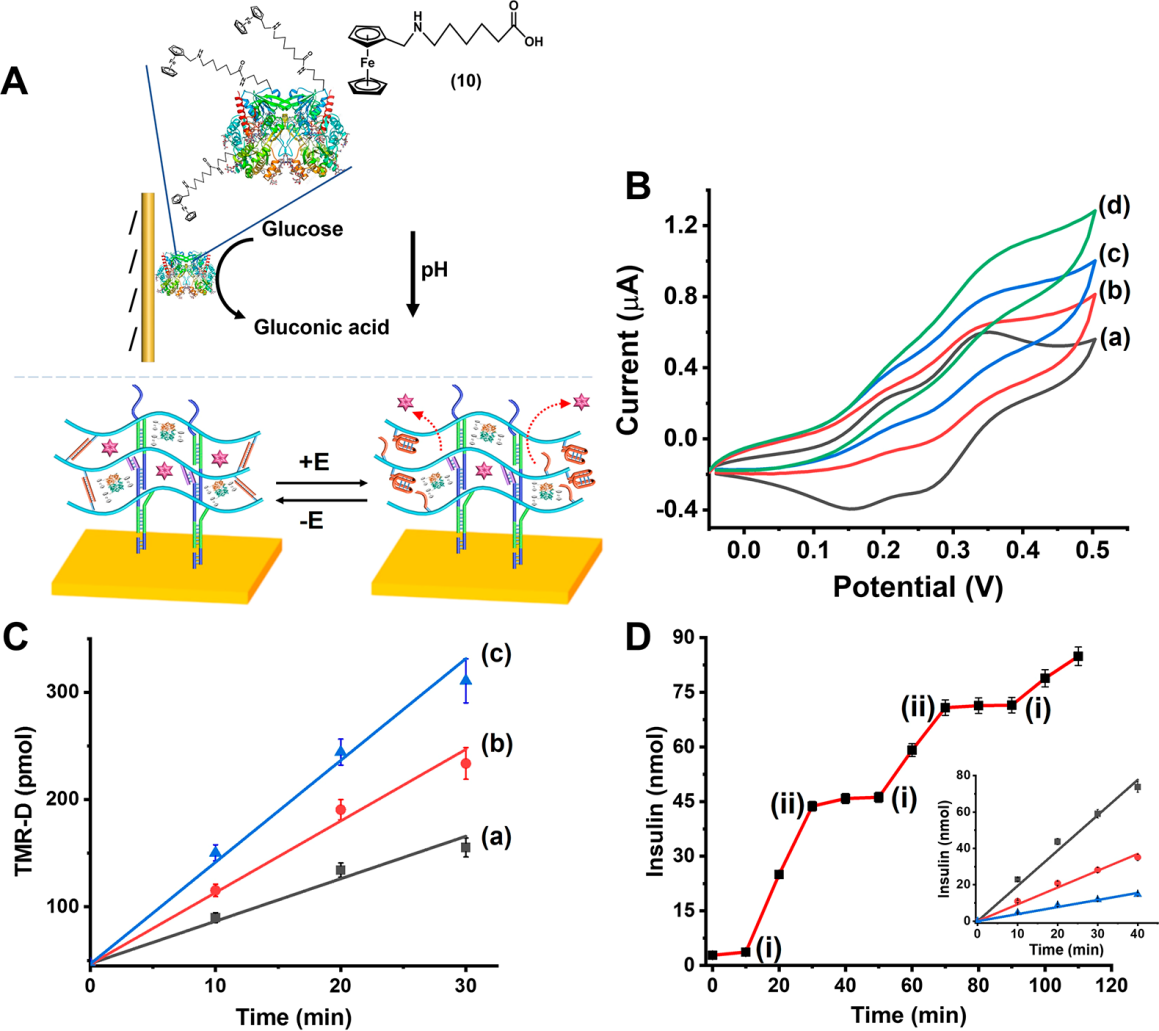
(A) Integrated electrically contacted pH-responsive hydrogel-functionalized Au electrode loaded with *N*-(ferrocenylmethyl)-6-aminohexanoic acid-modified GOx, 21 units, co-loaded with TMR-D, 3 μL of 25 mg/mL, or coumarin-functionalized insulin, 3 μL of 16 mM, for the bioelectrocatalyzed oxidation of glucose and the pH-stimulated release of the respective loads. (B) Cyclic voltammograms corresponding to the ferrocenyl-modified GOx-loaded hydrogel in the absence of glucose, (a), and in the presence of added glucose, (b) 100, (c) 250, and (d) 500 μM, scan rate of 10 mV sec^–1^. (C) Time-dependent release of TMR-D from the hydrogel carrier upon applying a fixed potential on the hydrogel-functionalized electrode, 0.4 V vs SCE, and in the presence of variable concentrations of glucose: (a) 100, (b) 250, and (c) 500 μM. (D) Switchable potential-induced release of coumarin-labeled insulin from the hydrogel-modified electrode carrier, in the presence of glucose, 250 μM. At point (i), the electrode is subjected to a potential of *E* = 0.4 V vs SCE. At point (ii), the potential is removed from the electrode. Inset: time-dependent release of coumarin-labeled insulin from the hydrogel carrier upon applying a fixed potential of 0.4 V vs SCE on the hydrogel-functionalized electrode, in the presence of variable glucose concentrations: (a) 100, (b) 250, and (c) 500 μM. All electrochemical experiments were performed in a HEPES buffer (10 mM, pH = 7.4, NaCl 50 mM, MgCl_2_ 5 mM). Error bars for the different experiments are derived from *N* = 4 experiments.

The electrochemically controlled, glucose-dictated, release of insulin has, certainly, potential practical applications for the future development of autonomous bioelectronic devices for controlling diabetes (bioelectronic “artificial pancreas”).^128^ To reach such goals, the elimination of diffusional electron mediators activating the release of insulin is, however, essential. Toward this goal, we designed an integrated insulin-loaded, pH-responsive, hydrogel-functionalized electrode loaded with an electrically wired glucose oxidase biocatalyst, as shown in Figure 6A. Glucose oxidase, GOx, was modified with *N*-(ferrocenylmethyl)-6-aminohexanoic acid, (**10**). The average loading of the enzyme corresponds to 12 ferrocenes per enzyme, and the ferrocene-functionalized GOx (Fc-GOx) revealed ca. 80% activity of the native enzyme. A gold electrode was functionalized with the promoter strand, (**6**), and a mixture of the polymers P_A_ and P_B_ that included the Fc-GOx biocatalyst, (3 μL of 50 mg/mL), deposited on the electrode, was polymerized on the electrode surface, using the promoter-induced HCR, to yield the hydrogel film consisting of the Fc-GOx-loaded, (**1**)/(**2**) + (**3**) and (**4**)/(**5**) cross-linked hydrogel matrix. Figure 6B depicts the cyclic voltammograms of the resulting Fc-GOx-loaded pH-responsive hydrogel-functionalized electrode in the absence of glucose, curve (a) and in the presence of variable concentrations of glucose, curves (b), (c), and (d). In the absence of glucose, two quasi-reversible waves of the ferrocene relay units, at *E* = 0.18 V (low intensity) and *E* = 0.3 V (higher intensity) vs SCE, are observed. The two redox waves are attributed to two different orientations of the ferrocene units linked to the GOx biocatalyst. In the presence of glucose, bioelectrocatalytic anodic currents are generated by the redox-relay units being oxidized at 0.4 V, implying that these relay units effectively wire the biocatalyst toward the oxidation of glucose. The bioelectrocatalytic currents are controlled by the concentration of glucose and as the concentration of glucose increases, the electrocatalytic anodic currents are intensified. Thus, the pH-stimulated stiffness changes of the hydrogel matrix, as a result of acidification of the hydrogel, are anticipated to be controlled by the concentrations of glucose. Indeed, microindentation experiments support the glucose-guided stiffness changes of the hydrogel matrix by the Fc-GOx-loaded hydrogel-modified electrode, see Figure S13, Supporting Information. Accordingly, the Fc-GOx-loaded pH-responsive hydrogel cross-linked by the permanent (**1**)/(**2**) + (**3**) and pH-responsive (**4**)/(**5**) bridges was co-loaded with TMR-Dextran, as a drug model, or with coumarin-labeled insulin, and the glucose-driven bioelectrocatalyzed release of the loads from the pH-responsive hydrogel was examined. Figure 6C depicts the time-dependent release of the TMR-D from the Fc-GOx-loaded, integrated, pH-responsive (**1**)/(**2**) + (**3**) and (**4**)/(**5**) cross-linked electrode, loaded with the TMR-D, upon applying a potential corresponding to 0.4 V vs SCE on the electrode, in the presence of variable concentrations of glucose. As the concentration of glucose is higher, the release rates of TMR-D are enhanced. Control experiments revealed that in the absence of glucose and applying the potential on the electrode, or in the presence of glucose and in the absence of applied potential, only trace amounts of TMR-D are released. These results are consistent with the Fc-GOx bioelectrocatalyzed oxidation of glucose, leading to the acidification of the hydrogel assembly and the formation of a lower stiffness hydrogel matrix that allows the release of load. As the concentration of glucose increases, the bioelectrocatalyzed oxidation of glucose is enhanced, resulting in increased pH changes, lowering the stiffness of the hydrogel, and enhancing rates of release of the load. (For the potential-induced cyclic switched-on and switched-off release of the TMR-dextran model load from the integrated Fc-GOx hydrogel assembly see Figure S14, Supporting Information). In the next step, the potential-induced switchable release of the coumarin-labeled insulin, in the presence of glucose, was demonstrated, Figure 6D. Subjecting the potential corresponding to 0.4 V vs SCE on the electrode triggers “ON” the release of insulin. Switching-off the potential results in the rapid neutralization of the hydrogel, its transition to the higher stiffness state, and the blockage of the release of insulin. By the cyclic ON/OFF switching of the potential on the electrode, the reversible switched-on and switched-off release of insulin is demonstrated. The rates of the bioelectrocatalyzed release of coumarin-labeled insulin are controlled, as expected, by the concentrations of glucose, as shown in Figure 6D, inset. As the concentration of glucose increases, the degree of acidification of the hydrogel increases and the stiffness of the hydrogel decreases, resulting in the enhanced release of load.

**Figure 7:**
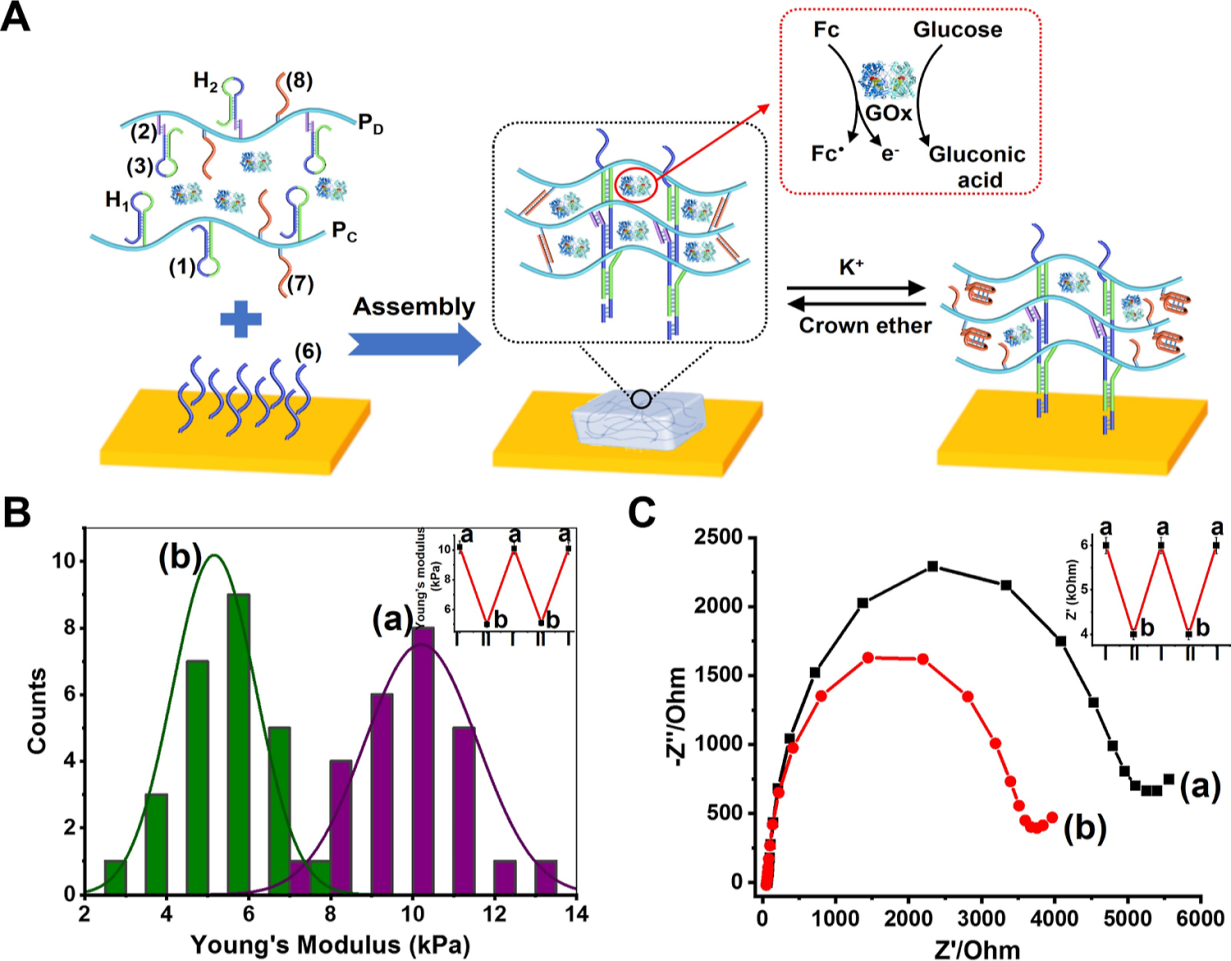
(A) Schematic assembly of the G-quadruplex/crown ether stiffness switchable hydrogel loaded with GOx. The control over the stiffness of the hydrogel guides the switchable ferrocenemethanol-mediated bioelectrocatalyzed oxidation of glucose. (B) Young’s modulus histograms corresponding to: (a) higher stiffness hydrogel cross-linked by the (**1**)/(**2**) + (**3**) duplexes and the (**7**)/(**8**) bridges, where (**7**) is the G-rich strand. (b) Lower stiffness K^+^-ion-treated hydrogel-modified electrode cross-linked by only the (**1**)/(**2**) + (**3**) bridges. Inset: switchable Young’s modulus of the G-quadruplex/crown ether hydrogel. (C) Faradaic impedance spectra corresponding to: (a) higher stiffness hydrogel-modified electrode cross-linked by the (**1**)/(**2**) + (**3**) and (**7**)/(**8**) duplex bridges. (b) Lower stiffness hydrogel generated in the presence of K^+^-ion where the hydrogel is cross-linked by only (**1**)/(**2**) + (**3**) bridges. Inset: switchable interfacial electron-transfer resistances in the absence/presence of crown ether. Error bars evaluated from *N* = 3 experiments.

The basic concepts introduced by the present study, where the bioelectrocatalytic functions of an enzyme (e.g., GOx) were switched by the stiffness-controlled permeability of the enzyme substrate and an electron mediator through a stimuli-responsive hydrogel matrix, were further demonstrated by integration of the GOx biocatalyst and switching its electrocatalytic function by means of K^+^-ion/crown ether stimuli-responsive hydrogel matrix, as shown in Figure 7A. The glucose oxidase was integrated in a G-quadruplex-responsive polyacrylamide hydrogel matrix. The promoter (**6**) was assembled on an Au electrode surface. The two polymer mixtures, P_C_ and P_D_, where P_C_ is functionalized with a hairpin, (**1**), and the nucleic acid (**7**), which are capable of forming, in the presence of K^+^ ion, the K^+^-ion-stabilized G-quadruplex, and P_D_ is modified with the hairpin, (**2**)+(**3**), and further functionalized with the tether, (**8**), complementary to (**7**), were deposited on the promoter-modified electrode, together with GOx (4 μL of 50 mg/mL). The promoter-induced hybridization chain reaction (HCR) resulted in the GOx-loaded hydrogel matrix, surface area 8.5 mm^2^, GOx loading 32 units, which is cooperatively cross-linked by the permanent duplexes (**1**)/(**2**) + (**3**) and the stimuli-responsive duplexes (**7**)/(**8**). In the presence of K^+^ ions, the duplex (**7**)/(**8**) is separated through the reconfiguration of the strand (**7**) into the K^+^-ion-stabilized G-quadruplex. Thus, the hydrogel cooperatively stabilized by the duplexes (**7**)/(**8**) and (**1**)/(**2**) + (**3**) is anticipated to reveal higher stiffness, whereas the separation of the duplex (**7**)/(**8**) yields a lower stiffness hydrogel. Treatment of the lower stiffness hydrogel with 18-crown-6-ether, CE, leads to the separation of the K^+^-ion-stabilized G-quadruplex and to the reassembly of the higher stiffness hydrogel. The reversible control over the stiffness of the hydrogel is, then, anticipated to guide the switchable activation of the bioelectrocatalytic functions of the GOx-loaded hydrogel-modified electrode; while in the higher stiffness hydrogel state, the permeability of the ferrocenemethanol electron mediator and the glucose substrate are hindered, leading to lower bioelectrocatalysis, the K^+^-ion triggered reconfiguration of the hydrogel into the G-quadruplex, yields the lower stiffness hydrogel, enhance the permeability of the electron mediator/glucose substrate, leading to enhanced bioelectrocatalysis. By cyclic treatment of the electrode with K^+^ ions and CE, the bioelectrocatalyzed oxidation of glucose is switched between lower and higher bioelectrocatalytic activities.

Figure 7B depicts the histograms of the Young’s moduli of the higher stiffness hydrogel cooperatively cross-linked by the (**1**)/(**2**) + (**3**) and (**7**)/(**8**) in the presence of CE, curve (a), revealing a Young’s module of ca. 10 kPa and the lower stiffness hydrogel upon K^+^-ion-triggered separation of the duplex (**7**)/(**8**) and formation of the G-quadruplexes, curve (b), revealing lower Young’s modulus corresponding to ca. 5 kPa. The Young’s modulus values of the hydrogel can be switched by the repeated application of K^+^-ions and crown ether for, at least, five cycles. Nevertheless, the switching efficiency decreases as the number of cycles increases due to the partial mechanical degradation of the hydrogel matrix upon the indentations. The control over the stiffness properties of the hydrogel matrix in the presence of K^+^-ions/CE is further supported by Faradaic impedance measurements, as shown in Figure 7C. The higher stiffness hydrogel cross-linked by the duplexes (**7**)/(**8**) and (**1**)/(**2**) + (**3**) reveals an interfacial electron-transfer resistance of ca 6.1 kΩ, as shown in Figure 7C, curve (a), whereas the interfacial electron-transfer resistance of the lower stiffness hydrogel, consisting of the K^+^-ion-stabilized G-quadruplex bridged by the duplexes (**1**)/(**2**) + (**3**) only shows a lower stiffness corresponding to *R*_et_ ≈ 4.1 kΩ, as shown in curve (b).

**Figure 8:**
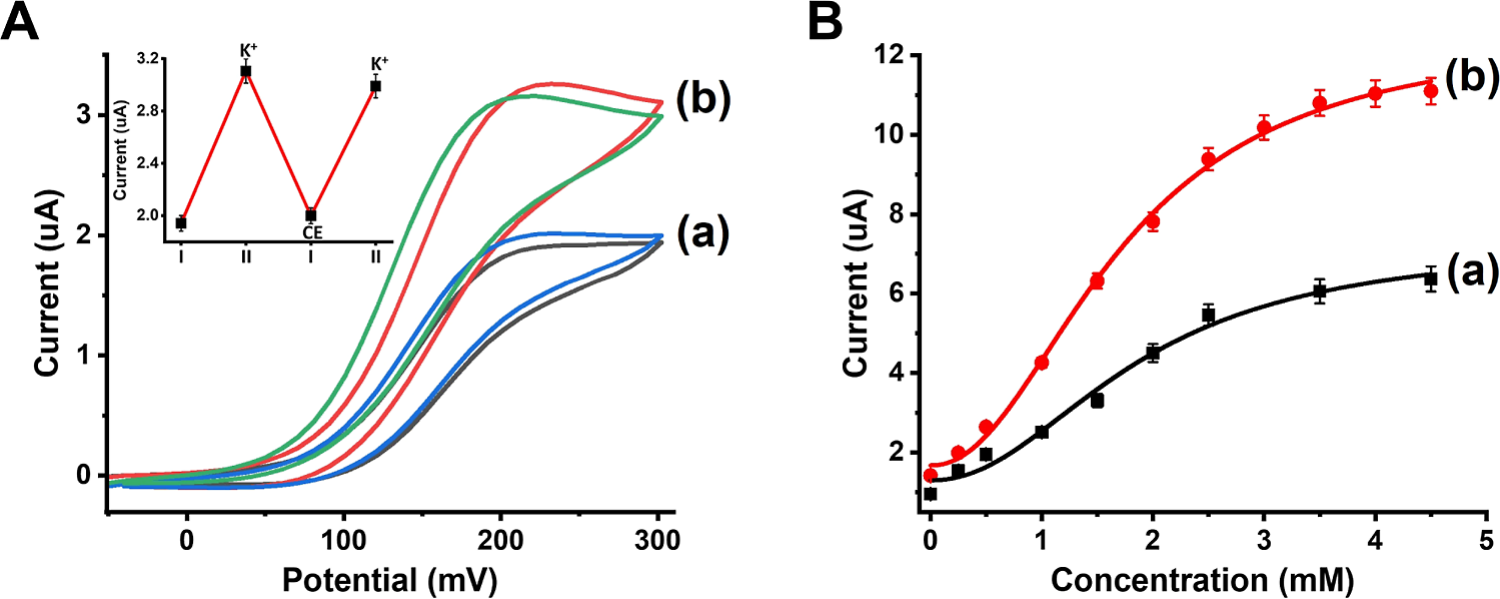
(A) Cyclic voltammograms corresponding to bioelectrocatalyzed oxidation of glucose by the G-quadruplex/crown ether-responsive GOx-loaded hydrogel: (a) in the presence of the higher stiffness hydrogel cross-linked by (**1**)/(**2**) + (**3**) and (**7**)/(**8**) bridges. (b) In the presence of the lower stiffness hydrogel cross-linked by (**1**)/(**2**) + (**3**) bridges only. Inset: switchable bioelectrocatalyzed oxidation of glucose by the G-quadruplex/crown ether responsive hydrogel. In all experiments, the hydrogel loaded with 3.2 units/mm^3^ GOx, ferrocenemethanol, 20 μM, is used as an electron transfer mediator and glucose, 500 μM. (B) Current responses of the GOx-loaded G-quadruplex/crown ether-responsive hydrogel-modified electrode in the presence of variable concentrations of glucose: (a) high stiffness hydrogel cross-linked by (**1**)/(**2**) + (**3**) and (**7**)/(**8**) duplex bridges. (b) Lower stiffness hydrogel cross-linked by only (**1**)/(**2**) + (**3**) duplex bridges. Error bars derived from *N* = 3 experiments.

The K^+^-ion/crown-ether switchable stiffness properties are reflected by the bioelectrocatalytic properties of the GOx-loaded K^+^-ion/CE-responsive hydrogel-modified electrode. In the presence of the stiffer hydrogel, the lower permeation efficacies of the electron mediator and substrate lead to less efficient bioelectrocatalyzed oxidation of glucose, reflected by a lower voltametric response, as shown in Figure 8A, curve (a). In turn, treatment of the electrode with K^+^-ions yields a lower stiffness hydrogel that enhances the permeation of the ferrocenemethanol electron mediator and of the glucose substrate, resulting in enhanced bioelectrocatalyzed oxidation of glucose, reflected by intensified anodic voltametric responses, as shown in Figure 8A, curve (b). By the cyclic treatment of the electrode with K^+^-ions and CE, the bioelectrocatalyzed oxidation of glucose is switched between high and low states, as shown in Figure 8A, inset. The switching process leads, however, to waste product K^+^-ion/crown ether complexes. The fact that the hydrogel framework is confined to the electrode allows one to rinse and wash-off the waste product after several switching cycles. We find that rinsing the electrode after three operation cycles allows one to switch the bioelectrocatalytic functions of the hydrogel matrix for 10 cycles with no significant degradation effect, as shown in Figure S15. The bioelectrocatalytic currents generated by the K^+^-ion/CE-responsive GOx-loaded electrode, in the presence of variable concentrations of glucose, are depicted in Figure 8B. The amperometric responses of the GOx-modified electrode are intensified as the concentration of glucose increases. The amperometric responses of the stiffer hydrogel electrode are, however, ca. 2-fold lower as compared to the lower hydrogel stiffness electrode, curve (a) vs curve (b), which is consistent with the enhanced permeation of the electron mediator and glucose into the lower stiffness hydrogel.

## Conclusions

The present study has introduced a versatile method to assemble stimuli-responsive, enzyme-loaded, hydrogel-functionalized electrodes. The triggered control over the stiffness of the hydrogel matrices switched the bioelectrocatalytic performance of the modified electrodes between enhanced bioelectrocatalytic functions (lower stiffness hydrogels) and reduced bioelectrocatalytic functions (higher stiffness hydrogels). Different physical methods to follow the switchable stiffness properties of the hydrogels associated with the electrode were introduced, and these included the characterization of the Young’s modulus values of the higher stiffness/lower stiffness hydrogels by microindentations and by following the effects of the hydrogel stiffness on the interfacial electron-transfer efficacies between the electrode and the permeating electron mediator using electrochemical means (Faradaic impedance spectroscopy and chronopotentiometric measurements). Two glucose oxidase (GOx)-loaded stimuli-responsive hydrogel-functionalized electrodes were introduced. One system included a GOx-loaded pH-responsive electrode that demonstrated the bioelectrocatalyzed oxidation of glucose through electrical contacting of the enzyme with the electrode by a diffusional electron mediator or by an electron mediator covalently linked to the GOx biocatalyst. The bioelectrocatalyzed oxidation of glucose by the GOx-integrated matrices led to acidification of the pH-responsive hydrogel and to the lowering of hydrogel stiffness. By co-immobilization of loads, particularly insulin, in the hydrogel matrices, the bioelectrocatalyzed, pH-stimulated, lowering of the hydrogel stiffness enabled the programmed controlled release of the loads (specifically insulin) from the hydrogel matrices. As the stiffness changes of the hydrogel matrices were controlled by the concentrations of glucose and the electrochemically switched states of the hydrogel between ON/OFF states, the programmed glucose concentration-guided and switchable ON/OFF releases of the loads from the hydrogel matrices were demonstrated. Besides the basic concept to control the stiffness of hydrogel by a bioelectrocatalytic process and to stimulate the controlled release of drugs, the relevance and potential of the system to develop autonomous devices for the controlled release of insulin for the management of diabetes (“artificial pancreas”)^128^ should be discussed. The switchable glucose concentration control of the stiffness of the pH-responsive hydrogel, and the accompanying “ON”/”OFF” release of insulin, suggest that the system could act as an autonomous bioelectronic device for the controlled temporal and dose-controlled release of insulin. Nonetheless, the translation of this bioelectronic system into a practical device needs further development. At present, the systems operate under anaerobic conditions and for practical applications, the functionalization of the electrode surface with oxygen-eliminating agents is essential. Indeed, in preliminary experiments, we introduced bilirubin oxidase and bilirubin as an oxygen removal biocatalytic system. Under these conditions, the electrode performance under aerobic conditions and argon were identical. However, for practical applications, the optimization, minimalization, and packaging of the bioelectronic device are essential steps to follow.

The second system demonstrated reversible and switchable stimuli-responsive bioelectrocatalytic functions of a K^+^-ion/crown ether GOx-loaded hydrogel-modified electrode. The electrocatalytic activities of hemin-G-quadruplex units^103^ pave the way to develop enzyme/hemin-G-quadruplex bioelectrocatalytic reactor sensing matrices. Moreover, besides the control of the stiffness of the hydrogels by means of bioelectrocatalytic processes and the application of the systems for controlled release, the systems pave the means to drive mechanical operations of the electrode matrices.^129−132^

## Experimental Section

Oligonucleotide sequences (5′ to 3′)

(**1**) /5Acryd/AAAAAAAAAAGGTGTTTAAGTTGGAGAATTGTACTTAAACACCTTCTTCT(**2**) /5Acryd/TTTGGACCGATGTTAGAGC(**3**) CAATTCTCCAACTTAAACTAGAAGAAGGTGTTTAAGTTGGGCTCTAACATCGGTCCAA(**4**) /5Acryd/AAAAACCCAATCCCAATCCCAATCCCT(**5**) /5Acryd/AAAAATGATTGTGATTGTGACCG(**6**) /5ThioMC6-D/TTTTTAGAAGAAGGTGTTTAAGTA(**7**) /5Acryd/AAAAAGGGTTAGGGTTAGGGTTAGGG(**8**) /5Acryd/AAAAACTCTAACCTTAATCCTAACTC

For the synthesis of acrylamide copolymer chains, electrode modification, hydrogel formation, catalysis, and controlled release of loads (see the Supporting Information).

## Supporting Information Available

Materials and instruments, experimental section, determination of ratio of acrylamide/acrydite-nucleic acids in the polymers, determination of molecular weight of acrydite-nucleic acid-modified polymers, SEM image, ^1^H NMR and DOSY NMR spectra, microindentations, time-dependent fluorescence changes upon the release of the loads from the hydrogel matrices, and calibration curves (PDF)
