# Supplementary material for: Stimuli-Responsive DNA-Based Hydrogels on Surfaces for Switchable Bioelectrocatalysis and Controlled Release of Loads
Source: ACS Appl Mater Interfaces. 2023 Jul 21;15(30):37011–25. doi: 10.1021/acsami.3c06230 (PMC10401574; doi:10.1021/acsami.3c06230)
Supplement: Supplementary file 1 — am3c06230_si_001.pdf [file am3c06230_si_001.pdf]

Supporting Information

Stimuli-Responsive DNA-Based Hydrogels on Surfaces for Switchable Bioelectrocatalysis and Controlled Release of Loads

Michael Fadeev, Gilad Davidson-Rozenfeld, Zhenzhen Li, Itamar Willner*

The Institute of Chemistry, The Center for Nanoscience and Nanotechnology, The Hebrew University of Jerusalem, Jerusalem 91904, Israel.

*Email: Itamar.willner@mail.huji.ac.il

Materials

Acrylamide, 2-Hydroxy-4′-(2-hydroxyethoxy)-2-methylpropiophenone (Irgacure2959), tris(2-carboxyethyl)phosphine) (TCEP), 4-(2-hydroxyethyl)-1-piperazineethanesulfonic acid (HEPES), 2-Amino-2-(hydroxymethyl)-1,3-propanediol (TRIS), Magnesium chloride, Sodium chloride, Potassium chloride, 18-Crown-6-ether, D-(+)-glucose, Glucose Oxidase from Aspergillus Niger, Tetramethylrhodamine dextran (TMR-D), Insulin, and Coumarin were purchased from Sigma-Aldrich. Freeze-dried oligonucleotides were purchased from Integrated DNA Technologies (Table S1). DNA stock solutions were prepared in ultrapure water purified by a NANOpure Diamond instrument (Barnstead International, Dubuque, IA, USA).

Instruments

UV/vis absorption spectra were recorded with a temperature-controlled UV-2401PC spectrophotometer (Shimadzu, Japan).

Fluorescence spectra were recorded with a Cary Eclipse Fluorometer (Varian Inc.).

Mechanical testing of gels was performed on the Piuma Nanoindenter (Optics11, Amsterdam, NL). Stiffness: 0.025 N/m; tip radius: 8.5 μm; model: Oliver & Pharr.

SEM images were taken with the Extra High Resolution Scanning Electron Microscope Magellan (TM) 400L with the following microscope settings: 2 kV, 13 pA. The hydrogel sample on Au-coated glass slide was frozen by immersing it in liquid nitrogen, followed by sublimation under high vacuum.

The ^{1}H-NMR and the diffusion-ordered NMR spectroscopy (DOSY) spectra were recorded on a Bruker DRX 400 MHz and a Bruker Ultrashield Plus 500 MHz spectrometer.

All electrochemical measurements were carried out using a PC-controlled (Autolab GPES/FRA software) potentiostat/galvanostat (Autolab, ECO Chemie, Netherlands).

Table S1 – Oligonucleotide sequences (5' to 3')

**(1)** /5Acryd/AAAAAAAAAAGGTGTTTAAGTTGGAGAATTGTACTTAAACACC TTCTTCT
**(2)** /5Acryd/TTTGGACCGATGTTAGAGC
**(3)** CAATTCTCCAACTTAAACTAGAAGAAGGTGTTTAAGTTGGGCTCTAA CATCGGTCCAA
**(4)** /5Acryd/AAAAACCCAATCCCAATCCCAATCCCT
**(5)** /5Acryd/AAAAATGATTGTGATTGTGACCG
**(6)** /5ThioMC6-D/TTTTTAGAAGAAGGTGTTTAAGTA
**(7)** /5Acryd/AAAAAGGGTTAGGGTTAGGGTTAGGG
**(8)** /5Acryd/AAAAACTCTAACCTTAATCCTAACTC

(9) Ferrocenemethanol

(10) N-(ferrocenylmethyl)-6-aminohexanoic acid, see ^{1}H-NMR, Figure S1.

The chemical was prepared according to the previously published protocol[1]

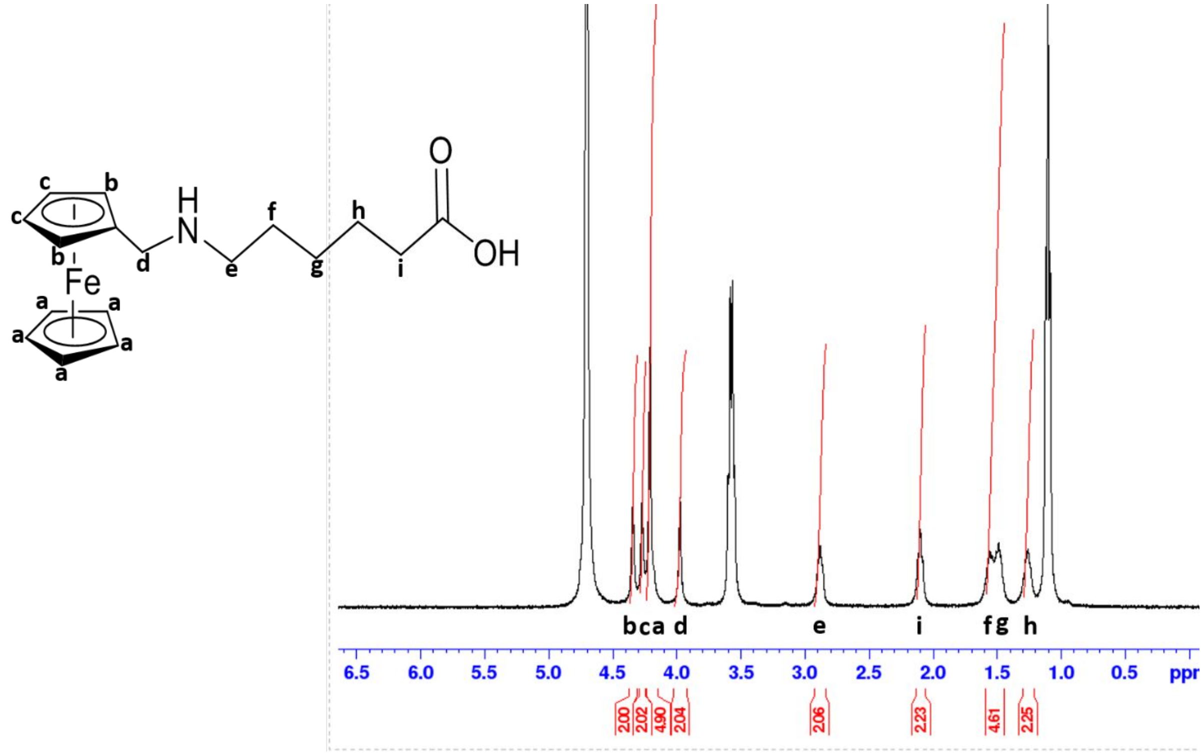

Figure S1. ^{1}H-NMR spectrum corresponding to N-(ferrocenylmethyl)-6-aminohexanoic acid in D2O.

Synthesis of the Acrylamide Copolymer Chains

As the acrydite modification is only available on the 5' end of DNA, H_1 **(1)** was modified directly while H_2 **(3)** was hybridized to a short tether strand **(2)** that itself was modified with acrydite. The acrydite-modified oligonucleotides were used to synthesize the copolymer chains. To solution consisting of 0.75 mM acrydite-modified oligonucleotide and 0.18 mM acrylamide was added 0.4% of the initiator (prepared as 17 mg of 2-Hydroxy-4'-(2-hydroxyethoxy)-2-methylpropiophenone in 100 μL of DMSO). After 10 minutes of nitrogen bubbling, the sample was exposed to UV light (λ = 365 nm) for 10 minutes and then incubated at 4°C for a time interval of 12 hours to form the copolymer chains. Finally, the samples were filtered through the Amicon (Millipore) spin filter unit to remove all the unreacted compounds. Polymers $\mathbf{P_A}$ and $\mathbf{P_C}$ were filtered through a MWCO 30 kDa column, while polymers $\mathbf{P_B}$ and $\mathbf{P_D}$ (before hybridization of **(3)**) were filtered through a MWCO 10 kDa column. Next, the purified polymers were subsequently subjected to freeze-drying, followed by dissolution in buffer to achieve a

concentration of 1 mg/20 μL. After determination of **(2)** concentration, **(3)** was added in a 1:1 molar ratio in the hybridization chain reaction (HCR) buffer (25 mM Tris, 25 mM $MgCl_2$, pH 7.4). Polymers were then incubated at 95°C for 5 minutes, followed immediately by a 30-minute ice incubation to ensure efficient closing of hairpins. Synthesis of polymers $\mathbf{P_A}$ and $\mathbf{P_B}$ containing the i-motif and the complementary strand was performed according to the procedure above, with the addition of sequences **(4)** and **(5)**, while synthesis of polymers $\mathbf{P_C}$ and $\mathbf{P_D}$ containing the G-quadruplex was performed with the addition of sequences **(7)** and **(8)**.

Determination of Molecular Weight of Acrylamide/Acrydite-Nucleic Acids

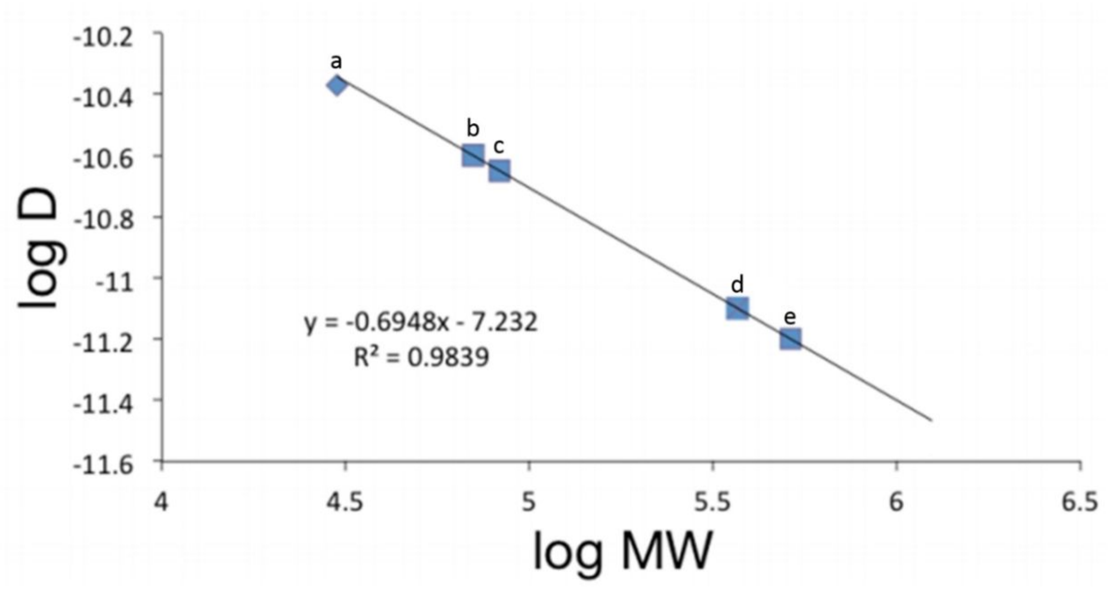

Figure S2. Calibration curve corresponding to the diffusion coefficients of a series of polymers of known average molecular weights: (a) Poly(acrylic acid) (MW ~ 35,000 Da) (b) Poly(acrylic acid), sodium salt (MW ~ 70,000 Da) (c) Poly(acrylic acid), sodium salt (MW ~ 85,000 Da) (d) Poly(acrylic acid) (MW ~ 345,000 Da) (e) Poly(acrylamide-co-acrylic acid) (MW ~ 520,000 Da). The diffusion coefficients of the respective polymers were derived by recording the DOSY spectrum of each of the polymers.

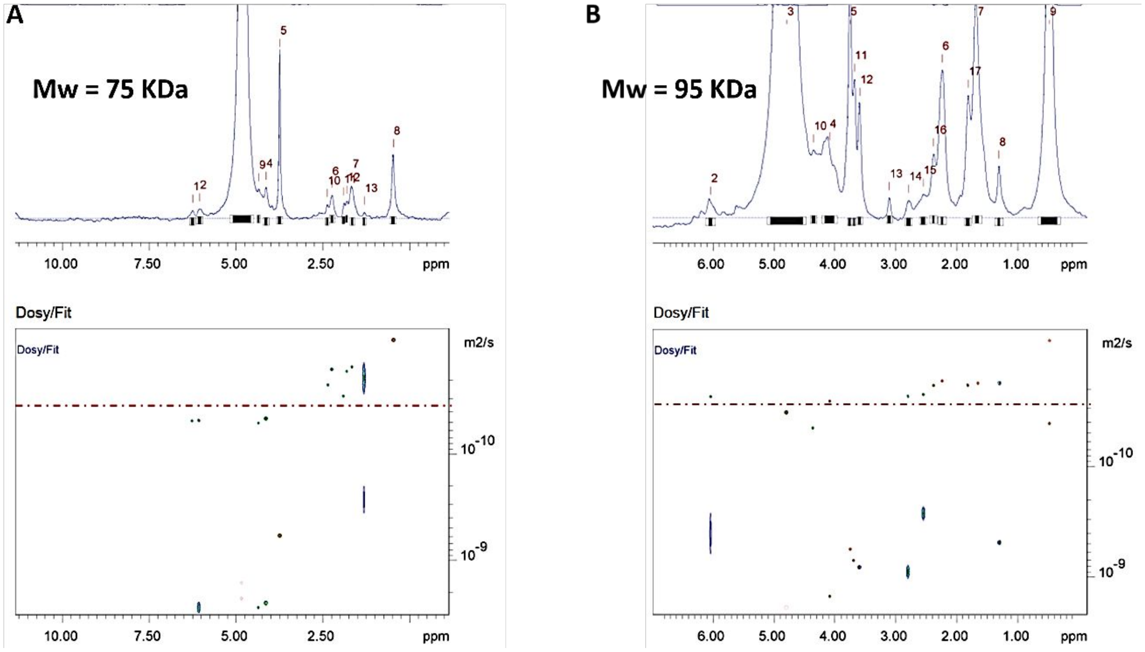

Figure S3. (A) The DOSY NMR spectrum of the PA polymer chain was used to estimate the molecular weight of the polymer (Mw = 75,000 Da). (B) The DOSY NMR spectrum of the PB polymer chain was used to evaluate the molecular weight of the polymer (Mw = 95,000 Da).

Determination of Ratio of Acrylamide/Acrydite-Nucleic Acids

To a solution containing the respective acrydite-modified nucleic acids, variable concentrations of pure acrylamide were added, and the absorption spectra of the different solutions were recorded. The increase in the absorbance at $\lambda = 200$ nm corresponded to the nonsubstituted polyacrylamide chains, while the absorbance at $\lambda = 260$ nm corresponded to the acrydite-modified nucleic acid units. An appropriate calibration curve corresponding to the molar ratio of the nucleic acids in the copolymer and the acrylamide monomer units was derived. Based on this calibration curve, the ratio of acrylamide/acrydite-nucleic acid in the different copolymers was evaluated spectroscopically (Figure S4).

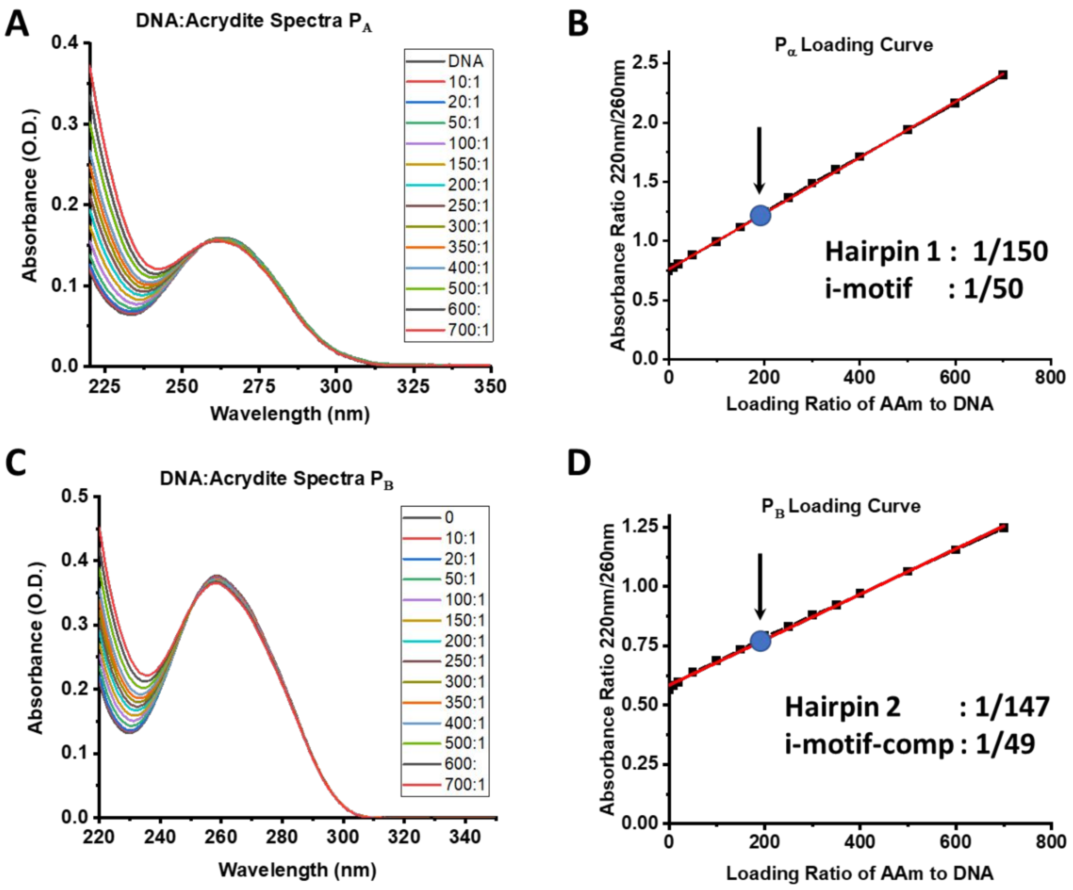

Figure S4. Determination of the loading of polyacrylamide polymer chains by the nucleic acids tethers: (A) Absorbance spectra of different concentrations of acrylamide in the presence of a constant concentration of the nucleic acids (1)+(4). (B) Calibration curve corresponding to absorbance ratio λ=200 nm/λ=260 nm as a function of the molar ratio of acrylamide to DNA. Arrow indicates the loading of the nucleic acid (1) on the polyacrylamide chains that corresponds to 1:150, while the loading of i-motif strand corresponds to 1/50. (C) Absorbance spectra of different concentrations of acrylamide in the presence of a constant concentration of the nucleic acids (2) +(5). (D) Calibration curve corresponding to absorbance ratio λ=200 nm/λ=260 nm as a function of the molar ratio of acrylamide to DNA. Arrow indicates the loading of the nucleic acid (2) on the polyacrylamide chains that corresponds to 1:147, while the loading of i-motif complementary strand corresponds to 1/49.

Modification of Gold Surface

A solution of 15 µM of **(6)** was mixed with a 150-fold excess of TCEP and incubated for 10 minutes in coating buffer (Tris, 25 mM, pH 7.4, $MgCl_2$, 5 mM). Gold-coated glass

slides were boiled in pure ethanol for 30 minutes, followed by immersion of slides in solution of **(6)**. Slides were incubated for 12 hours at room temperature and then washed with ultrapure water before use.

Gelation

Surface-integrated hydrogels were prepared by placing the polymer $\mathbf{P_B}$ or $\mathbf{P_D}$ (7.5 µL of 1 mg/20 µL) onto the gold surface coated by **(6)** and the polymer $\mathbf{P_A}$ or $\mathbf{P_C}$ (7.5 µL of 1 mg/20 µL) was added and mixed, correspondingly. Samples were then incubated for 12 hours at 4 ºC in a closed chamber with buffer reservoir to reduce evaporation of solution on surface. GOx (3 µL of 50 mg/mL) and TMR-D (3 µL of 25 mg/mL) or insulin (3 µL of 16 mM) were added to the polymer solution prior to gelation process.

The resulting hydrogel has a following dimensions:

m = 19.5 mg

V = 8.38 mm^3

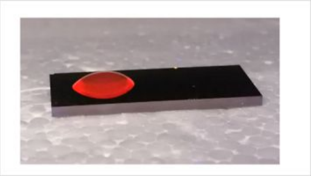

Thickness = 1 mm (measured with caliper).

GOx loading = 3.22 units/mm^3

Glucose oxidase activity was evaluated by ABTS assay[2]

The hydrogel dimensions can be controlled by the volume of polymerizing solution deposited on the surface and the concentration of the constituents in the polymerizing solution.

The insulin/GOx-fluorophore conjugates

The insulin and GOx -fluorophore conjugates were prepared as it was described previously[3]. GOx was modified with fluorescein isothiocyanate isomer I (FITC) (λ_{ex} = 490 nm; λ_{em} = 525 nm) and insulin was modified with 7-hydroxycoumarin-3-carboxylic acid succinimidyl ester (Coumarin) (λ_{ex} =360 nm; λ_{em} = 410 nm).

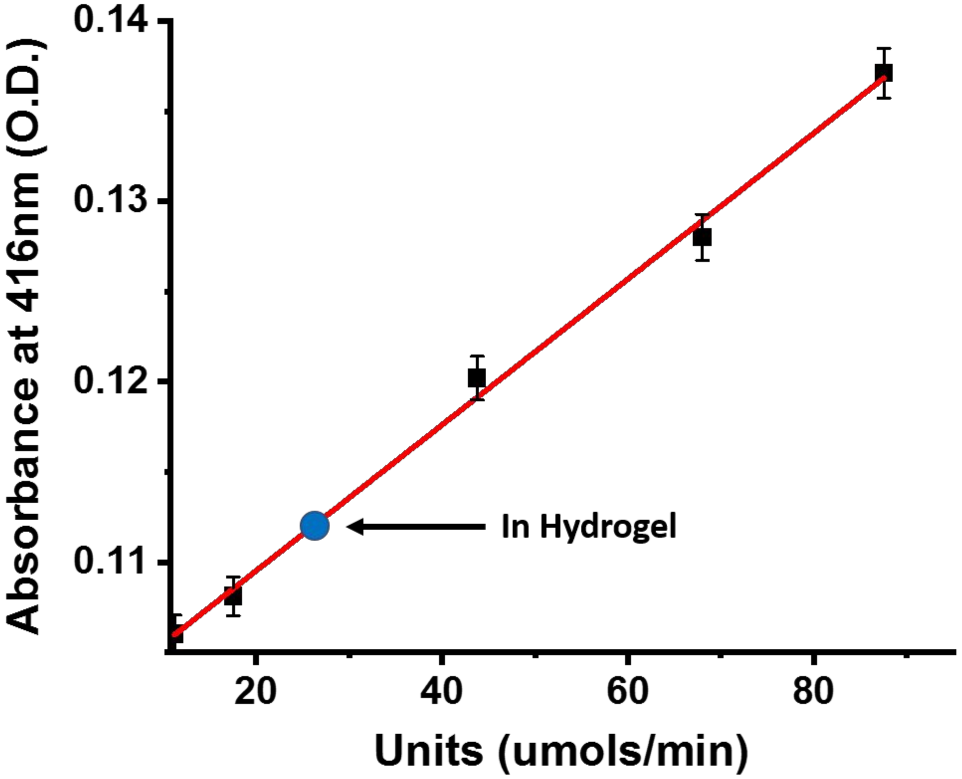

Figure S5. Calibration curve of glucose oxidase activity by ABTS assay. Arrow indicates the loading of the glucose oxidase incorporated into the hydrogel matrix.

Catalysis

Catalysis was performed by incubating the hydrogel-coated slide in buffer solution under following conditions:

1) The switchable biocatalytic oxidation of glucose and release of drugs from the i-motif-modified hydrogel matrix were performed under aerobic conditions in HEPES buffer (10 mM. pH=7.4, NaCl 50 mM, $MgCl_2$ 5 mM) by addition of different glucose concentrations.
2) The switchable bioelectrocatalytic oxidation of glucose and release of drugs from the i-motif-modified hydrogel matrix were performed under anaerobic conditions in HEPES buffer (10 mM. pH=7.4, NaCl 50 mM, $MgCl_2$ 5 mM) by applying

potential of E=0.35 V vs. SCE for diffusional system, or potential of E=0.4 V vs. SCE for integrated system and addition of different glucose concentrations.

3) The switchable bioelectrocatalytic oxidation of glucose by the G-quadruplex-modified GOx-loaded hydrogel matrix was performed under nitrogen in Tris buffer (25 mM. pH=7.4, $MgCl_2$ 20 mM) with the addition of 20 mM K^+, followed by addition of 18-crown-6-ether at 22 mM, and continued by adding 1.1x the concentration of previous component added (either K^+ or 18-crown-6-ether), by applying potential of E=0.4 V vs. SCE and addition of different concentrations of glucose.

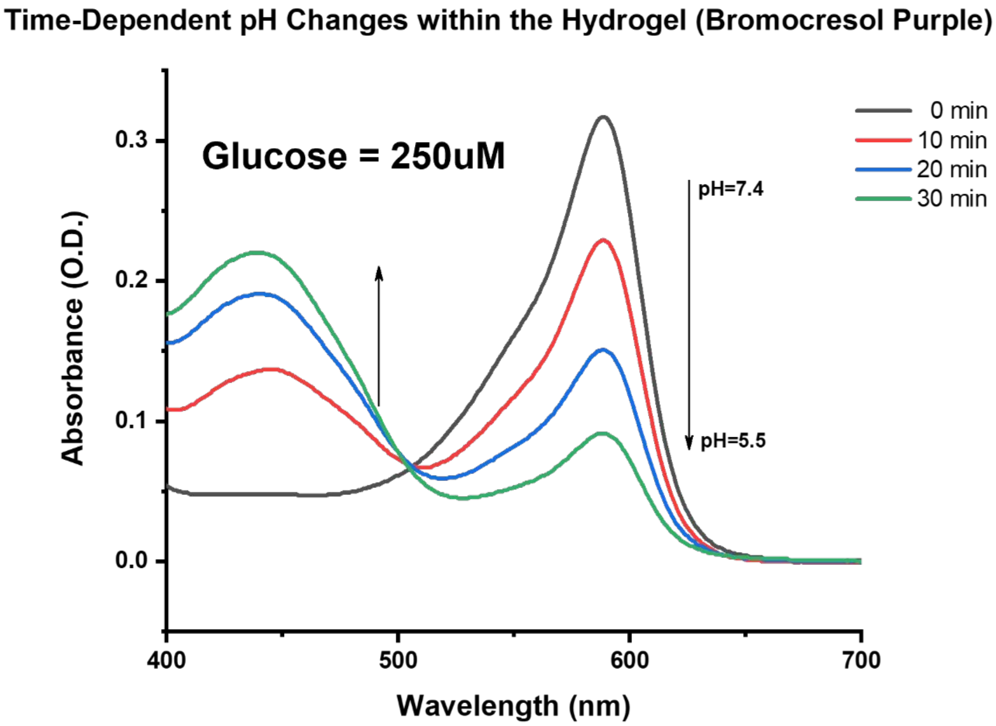

Figure S6. Time-dependent changes in absorbance reflect the pH changes occurring inside the hydrogel. To monitor these changes, the pH-sensitive dye bromocresol purple (5′,5″-dibromo-o-cresolsulfophthalein) was used, which has a pK_A = 6.3 and exhibits a yellow color at pH = 5.5 and violet color above pH = 7. The glucose concentration was 250 µM.

Time-dependent Faradaic impedance spectra

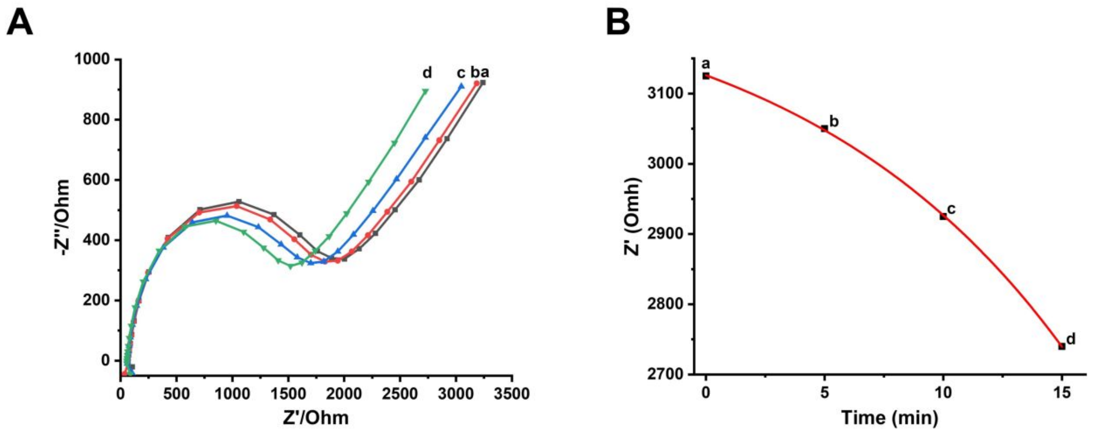

Figure S7. (A) Time-dependent Faradaic impedance spectra to track the dynamic transitions of the hydrogel between the high-stiffness and the low-stiffness states: a) Stiff hydrogel, b) After 5 minutes of biocatalysis, c) After 10 minutes of biocatalysis, d) After 15 minutes of biocatalysis. (B) Time-dependent changes in the electron transfer rate as a result of the biocatalyzed stiffness changes: a) Stiff hydrogel, b) After 5 minutes of biocatalysis, c) After 10 minutes of biocatalysis, d) After 15 minutes of biocatalysis.

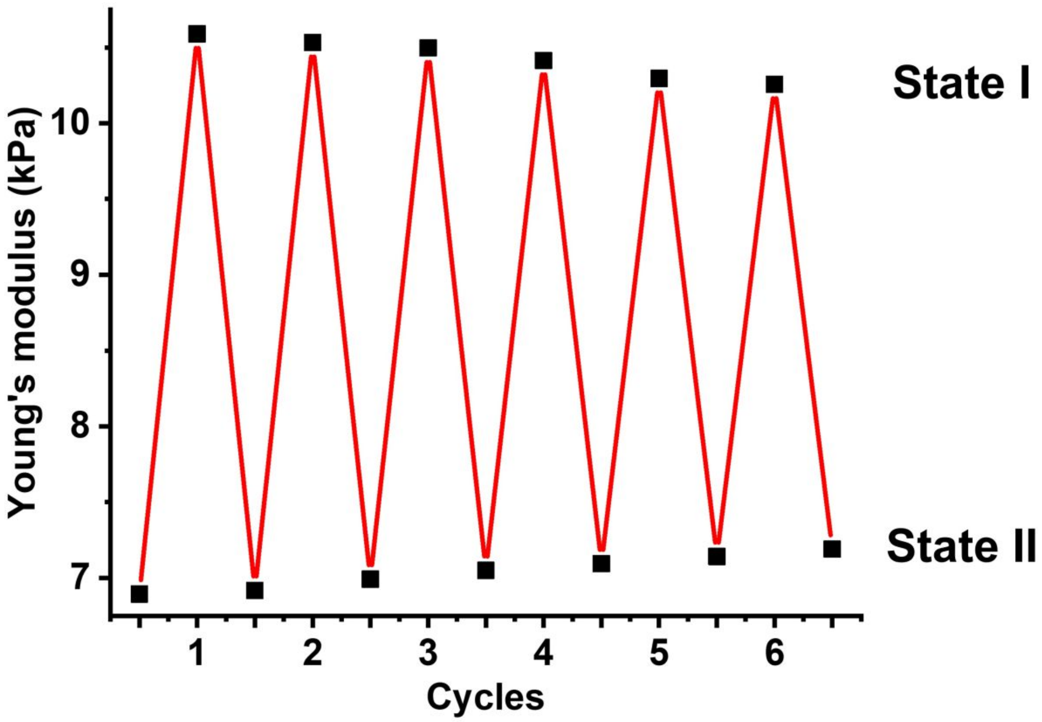

Figure S8. The Young's modulus values reversibly switched between a stiff hydrogel matrix (State I) and after subjecting the hydrogel to glucose (State II), across six cycles. The switching efficiency decreases with increasing the number of switching cycles attributed to the mechanical degradation of the hydrogel framework during the indentation process.

Calibration Curves for Loads

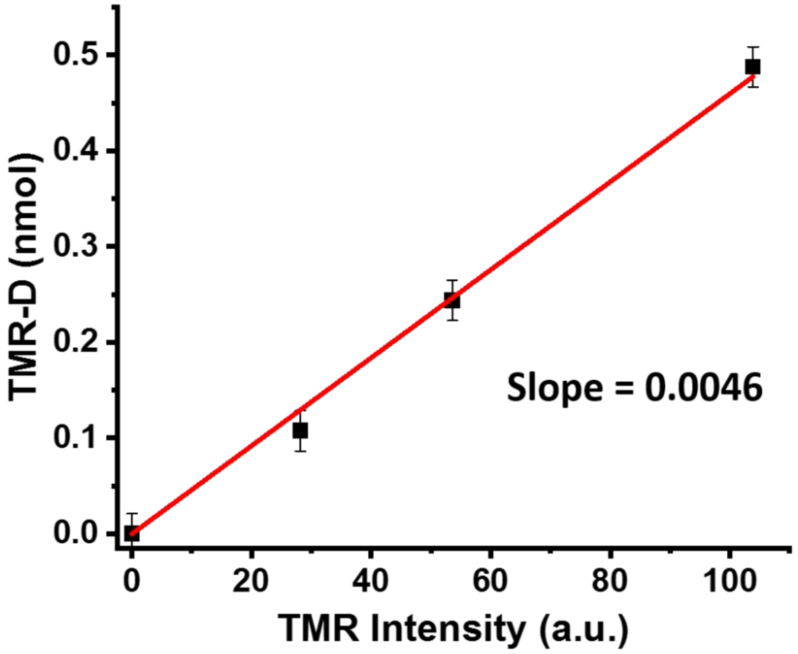

Figure S9. Calibration curve corresponding to Tetramethylrhodamine-modified dextran, TMR-D (λex = 546 nm; λem = 580 nm).

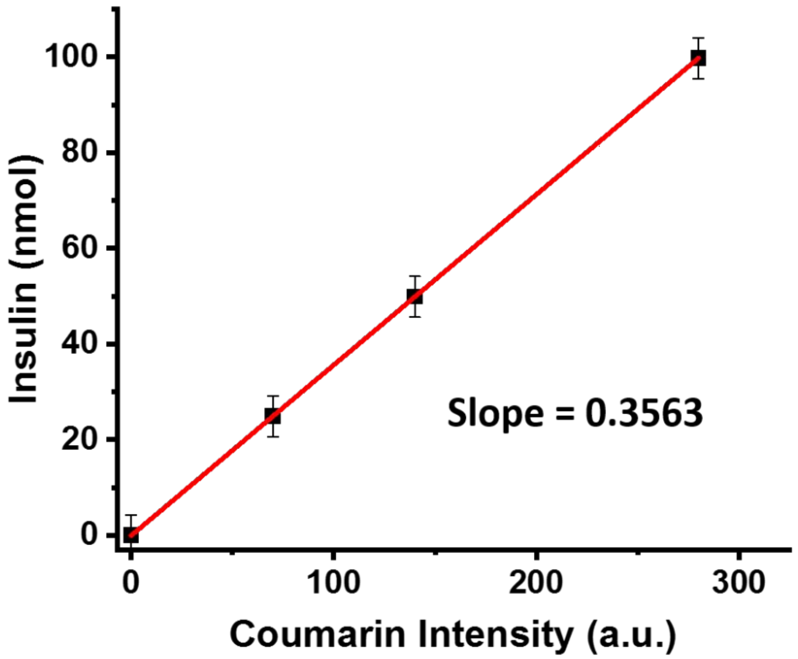

Figure S10. Calibration curves corresponding to 7-hydroxycoumarin-3-carboxylic acid N-succinimidyl ester (Coumarin)-modified insulin. (λex =360 nm; λem = 410 nm).

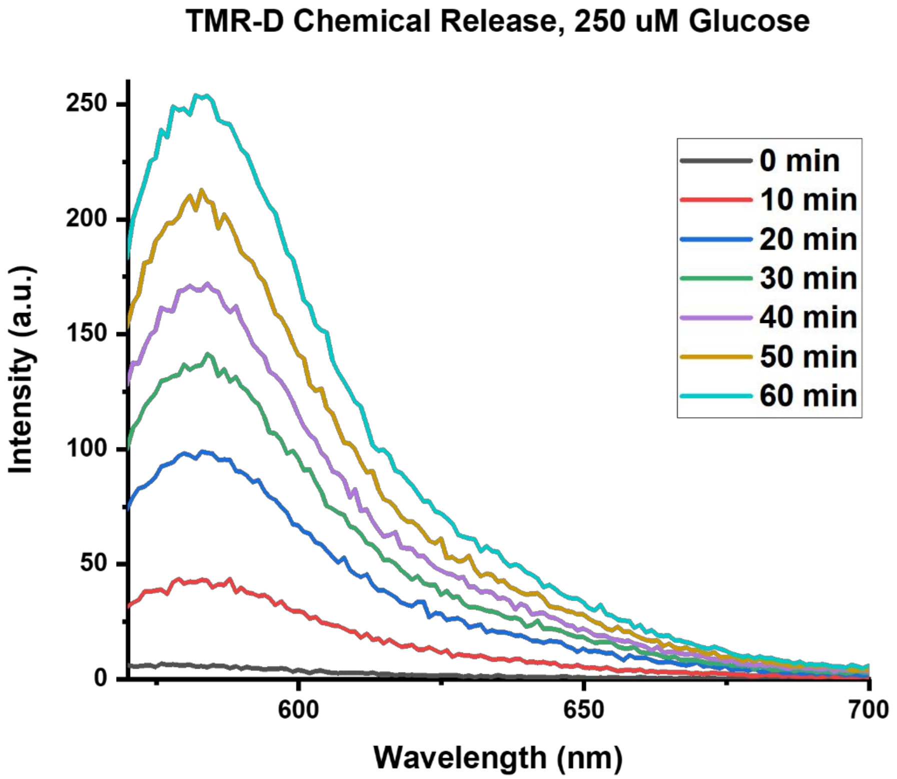

Figure S11. Triggered time-dependent release of TMR-D from i-motif-functionalized hydrogel matrix by applying constant potential of 0.4 V vs. SCE. Glucose concentration 250 µM.

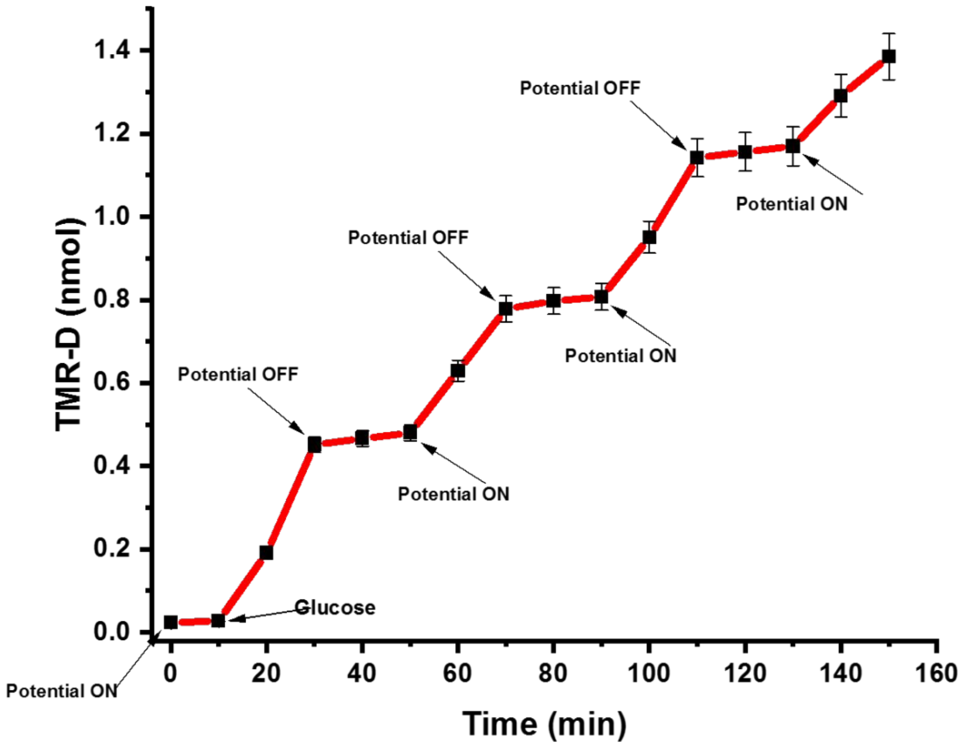

Figure S12. Switchable, potential-induced release of TMR-D from the GOx-functionalized pH-responsive hydrogel in the presence of glucose 100 µM. Subjecting the potential corresponding to 0.35 V vs. SCE on the electrode switches-on the bioelectrocatalyzed oxidation of glucose, acidification of the hydrogel and the release of TMR-D.

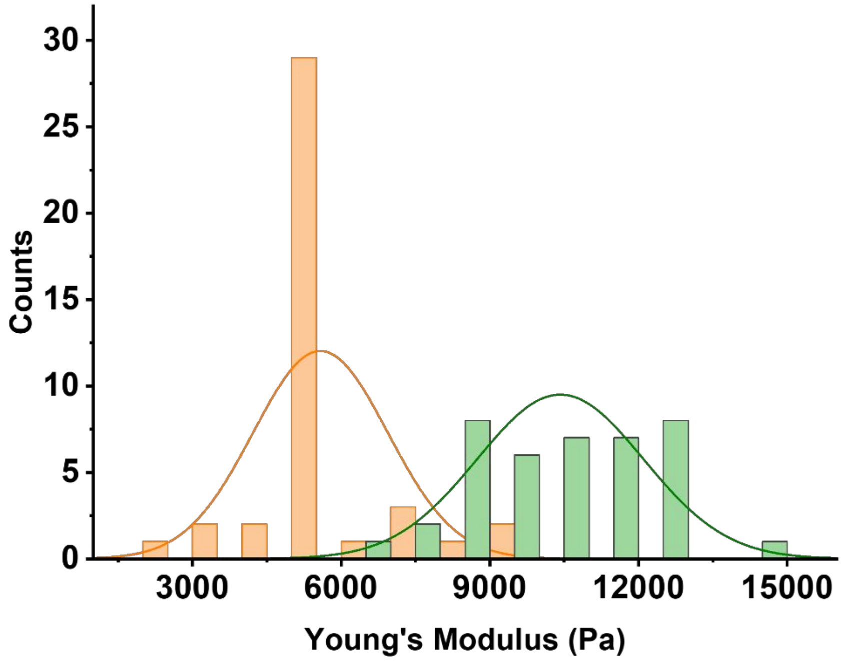

Figure S13. Microindentations experiment support the glucose-guided stiffness changes of the hydrogel matrix by the Fc-GOx-loaded hydrogel-modified electrode.

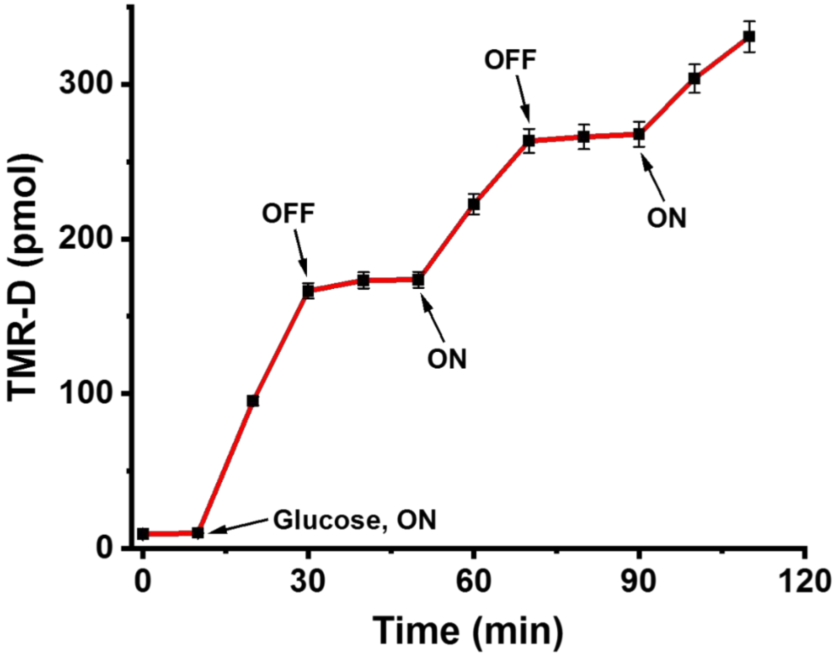

Figure S14. Switchable, potential-induced, time-dependent release of the TMR-D load from the GOx-functionalized ferrocene-modified pH-responsive hydrogel in the presence of glucose 250 µM. Subjecting the potential corresponding to 0.4 V vs. SCE on the electrode switches-on the bioelectrocatalyzed oxidation of glucose, acidification of the hydrogel and the release of TMR-D.

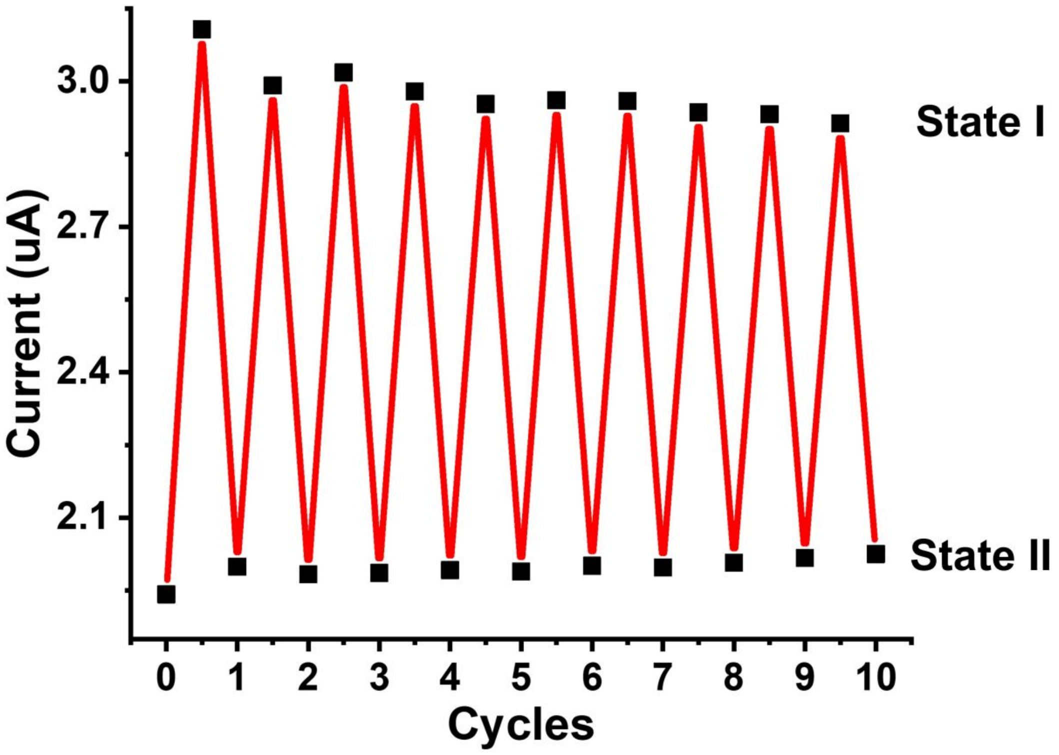

Figure S15. The bioelectrocatalytic functions of the hydrogel matrix were successfully switched between a hydrogel of lower stiffness, in the presence of K+-ions (State I) and a hydrogel of enhanced stiffness, in the presence of crown ether (State II). The reversible switching was monitored for 10 cycles, demonstrating consistent performance without a significant decrease.

References:

(1) Shoham, B.; Migron, Y.; Riklin, A.; Willner, I.; Tartakovsky, B. A Bilirubin Biosensor Based on a Multilayer Network Enzyme Electrode. *Biosens. Bioelectron.* **1995**, *10*, 341–352..

(2) Yee, Y. C.; Hashim, R.; Mohd Yahya, A. R.; Bustami, Y. Colorimetric Analysis of Glucose Oxidase-magnetic Cellulose Nanocrystals (CNCS) for Glucose Detection. *Sensors (Switzerland)* **2019**, *19*, 1–12.

(3) Hentz, N. G. Synthesis and Characterization of Insulin-Fluorescein Derivatives for Bioanalytical Applications. *Anal. Chem.* **1997**, *69*, 4994–5000.
